# OsCPK12‐OsATPD1 Module Regulates Photosynthetic Acclimation to Light Stress in Rice

**DOI:** 10.1002/advs.77268

**Published:** 2026-09-10

**Authors:** Beifang Wang, Aaron Chan, Yongyi Fan, Yanwu Wang, Pao Xue, Yingxin Zhang, Xiaodeng Zhan, Weixun Wu, Ping Yu, Daibo Chen, Junlin Fu, Yongbo Hong, Xihong Shen, Lianping Sun, Aike Zhu, Shihua Cheng, Qunen Liu, Liyong Cao

**Affiliations:** ^1^ Northern Center of China National Rice Research Institute Hangzhou China; ^2^ State Key Laboratory of Rice Biology and Breeding China National Rice Research Institute Hangzhou China; ^3^ Zhejiang Key Laboratory of Super Rice Research China National Rice Research Institute Hangzhou China; ^4^ Northern Rice Research Center of Bao Qing Shuangyashan China; ^5^ Zhejiang Guodao High‐Tech Seed Industry Co., Ltd China National Rice Research Institute Hangzhou China; ^6^ National Nanfan Research Institute (Sanya) Chinese Academy of Agricultural Sciences Sanya China

**Keywords:** OsAtpD1, OsCPK12, phosphorylation, photosynthetic acclimation

## Abstract

The synergy between light and dark reactions is the key to photosynthesis. This synergy not only helps plants achieve maximum photosynthetic efficiency but also plays a crucial role in photosynthetic protection. However, the mechanisms through which plants regulate this process remain unclear. In this study, we demonstrated that the calcium‐dependent protein kinase OsCPK12 is essential for mediating rice's responses to light intensity. The *oscpk12‐cr* mutant displayed light‐sensitive premature senescence. Specifically, light‐induced OsCPK12 expression targeted Ser8 on the chloroplast transit peptide of OsAtpD1, a subunit of ATP synthase, thereby promoting its transport into chloroplasts. *OsAtpD1^S8D^* overexpression in *oscpk12‐cr* rescued its premature senescence. ATPase activity and electron transport rate (ETR) were decreased in *oscpk12*‐*cr*, *oscpk12*‐*cr*/OE‐*OsAtpD1^S8A^*, and RNAi‐*OsAtpD1* plants. In contrast, *OsCPK12* overexpression in rice resulted in higher ATPase activity and ETR than in ZH8015. Overall, OsCPK12 phosphorylated OsAtpD1 to promote its chloroplast translocation, thereby maintaining ATP synthase activity and proton gradient (ΔpH) homeostasis and regulating photosynthetic acclimation, photoprotection, and repair under light stress. Our findings reveal the intrinsic mechanism by which the OsCPK12‐OsATPD1 module regulates ATP synthase activity and ETR efficiency, fine‐tuning photosynthetic acclimation to prevent light damage.

## Graphical abstract

OsCPK12 interacts and phosphorylates OsAtpD1 at Ser8 in its transit peptide. This phosphorylation promote its chloroplast translocation, thereby maintaining ATP synthase activity and proton gradient (ΔpH) homeostasis and regulating photosynthetic acclimation, photoprotection, and repair under light stress. OsCPK12‐OsAtpD1 module coordinately regulates photosynthetic acclimation by maintaining ATP synthase activity and ΔpH homeostasis, thereby enhance rice photosynthetic resilience under light stress.

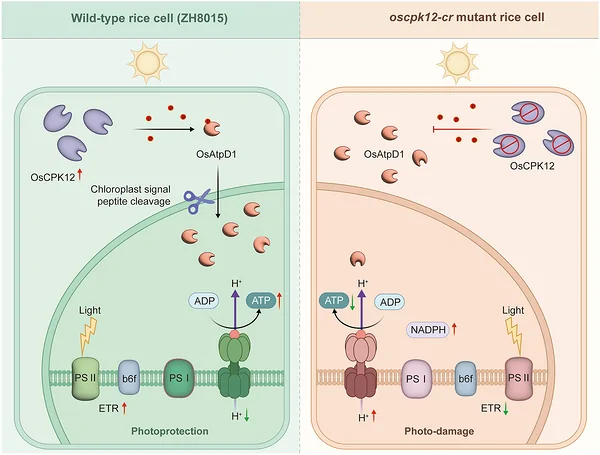

## 1 Introduction

Photosynthesis is the process by which green plants and other organisms convert light into chemical energy. Organic matter produced through photosynthesis accounts for approximately 90% of a crop's dry weight; therefore, enhancing photosynthetic efficiency is a pivotal objective for boosting crop yield [1]. The photosynthetic electron transport chain is primarily involved in linear electron transport (LET) from water to oxidized nicotinamide adenine dinucleotide phosphate (NADP^+^). At the heart of photosynthesis lies the intricate interplay between photosynthetic complexes, including Photosystem II (PSII), Photosystem I (PSI), the cytochrome b6f complex (Cytb6f), and adenosine triphosphate (ATP) synthase. The establishment of proton motive force (PMF) is a central event in photosynthetic energy conversion. The process begins with light absorption by PSII, during which water molecules are split, releasing oxygen, protons, and high‐energy electrons. These electrons are transferred to PSI through a series of carriers, including plastoquinone (PQ), Cytb6f, and plastocyanin (PC), where they are re‐energized by light and used to reduce NADP^+^ to nicotinamide adenine dinucleotide phosphate (NADPH). Concurrently, protons are pumped into the thylakoid lumen, creating a transmembrane proton gradient (ΔpH) and an electric potential (ΔΨ), which together establish the PMF, which is utilized by ATP synthase to produce ATP from Pi and ADP, completing the conversion of light energy into chemical energy [2, 3, 4].

In addition to ATP production, PMF also plays a central role in photosynthetic protection. It serves as a dynamic, central regulatory signaling network by monitoring its own intensity (primarily ∆pH) in real time and precisely activating various light‐protection mechanisms to ensure that photosynthesis not only efficiently captures light energy under fluctuating light (FL) conditions but also minimizes light damage to the greatest extent possible. ∆pH serves as a vital signal for photosynthetic regulation [5, 6, 7]. Excess light energy can damage the photosynthetic apparatus and reduce photosynthetic efficiency. The carbon reaction rate cannot match the light reaction under excess light energy, leading to the accumulation of ATP and NADPH, obstruction of the electron transport chain, and an abnormal increase in proton concentration, resulting in a significantly increased pH. A high pH slows the turnover rate of Cytb6f, thereby reversing the flow rate of the entire linear electron‐transfer chain. This prevents excessive electron influx into PSI and the generation of ROS, such as O_2_, and adjusts the PSI redox state to prevent photoinhibition [8, 9, 10, 11, 12, 13]. A high ∆pH is also a key environmental signal for triggering the induction of NPQ in PSII. This process is critical for dissipating excess light energy as thermal energy, thereby avoiding damage to the PSII reaction center [12, 14, 15, 16]. The pH also induces state transitions that balance the energy distribution between PSII and PSI. When PSII is overexcited and relatively starved, part of the PSII antenna protein LHCII is phosphorylated and migrates from the stacked grana to the non‐stacked stroma lamellae, binding to PSI. This process prevents PSII damage from excess energy and improves PSI efficiency [17, 18, 19]. PMF drives ATP synthesis, providing ATP for PSII repair. PMF is regulated by modulation of chloroplast ATP synthase, which creates an essential regulatory bridge between light reactions and subsequent metabolism [4, 20]. Chloroplast ATP synthase plays an important role in regulating photosynthetic electron flow in higher plants [9, 11, 21]. ATP synthase can adopt multiple conformational states, allowing it to fine‐tune the proton flow in response to changes in light intensity and metabolic demand. When chloroplast ATP synthase activity (g_H_^+^) is strongly repressed, resulting in lumen overacidification, it restricts assimilation capacity and linear electron flow [21]. For example, *Arabidopsis* mutants *hope2* and *cfq* exhibited a lower ∆pH compared to the wild‐type (WT) due to enhanced g_H_^+^, which led to excess electron flow from PSII to PSI, in turn leading to the production of ROS within PSI, and subsequently causing severe photodamage [9, 11]. Huang et al. (2018) [22] reported that chloroplast ATP synthase is essential for photoprotection and optimizing light‐use efficiency under FL conditions by coordinating with cyclic electron flow (CEF) to regulate PMF formation. However, the precise role of ATP synthase in regulating photosynthetic acclimation to light stress, as well as its specific molecular mechanism, remains unclear.

Calcium‐dependent protein kinases (CPKs) have distinct structural organizations. They feature an N‐terminal variable domain, followed by a serine/threonine kinase domain, an auto‐inhibitory junction domain, and a calmodulin‐like domain (CLD). The CLD contains four EF‐hand motifs capable of binding calcium ions (Ca^2+^) [23]. Subsequently, activated CPKs initiate downstream signaling events through phosphorylation, thereby modulating various cellular activities and responses [24]. CPKs are crucial for signaling under abiotic and biotic stress conditions [23, 24]. In our previous study, we characterized the rice mutant *oscpk12*, which exhibits leaf yellowing during the tillering stage. *OsCPK12* overexpression in the WT ZH8015 rice variety increased net photosynthetic rate (*P*_n_) and chlorophyll content [25]. However, the specific biochemical mechanism by which OsCPK12 modulates photosynthesis and light responses remains elusive. Here, we identified key regulatory roles of the OsCPK12‐OsAtpD1 module in this process. The loss of OsCPK12 function impairs ATP synthase activity, leading to lumen overacidification, altered photosynthetic electron transport (reduced ETR and qP, elevated NPQ and Y(NA)), NADPH accumulation, accelerated D1 protein degradation, and compromised photosynthetic repair capacity. Consequently, *oscpk12*‐*cr* mutants exhibited aggravated photodamage and premature senescence under high and FL conditions. We further showed that OsCPK12 interacts with OsAtpD1, a δ‐subunit of chloroplast ATP, and predominantly phosphorylates OsAtpD1 at Ser8 within the chloroplast transit peptide (Ctp). This phosphorylation is associated with efficient accumulation of OsAtpD1 in chloroplasts, which is crucial for maintaining electron transport, photosynthetic efficiency, and chloroplast ATP synthase activity in rice. *OsCPK12* overexpression confers on rice greater tolerance to high light intensity. Collectively, our data support the hypothesis that the OsCPK12‐OsAtpD1 molecular module promotes efficient photosynthesis and photoprotective responses in rice under high‐light (HL) stress.

## 2 Results

### 2.1 *OsCPK12* May be Involved in Regulating Photosynthetic Acclimation to Light Stress in Rice

**FIGURE 1:**
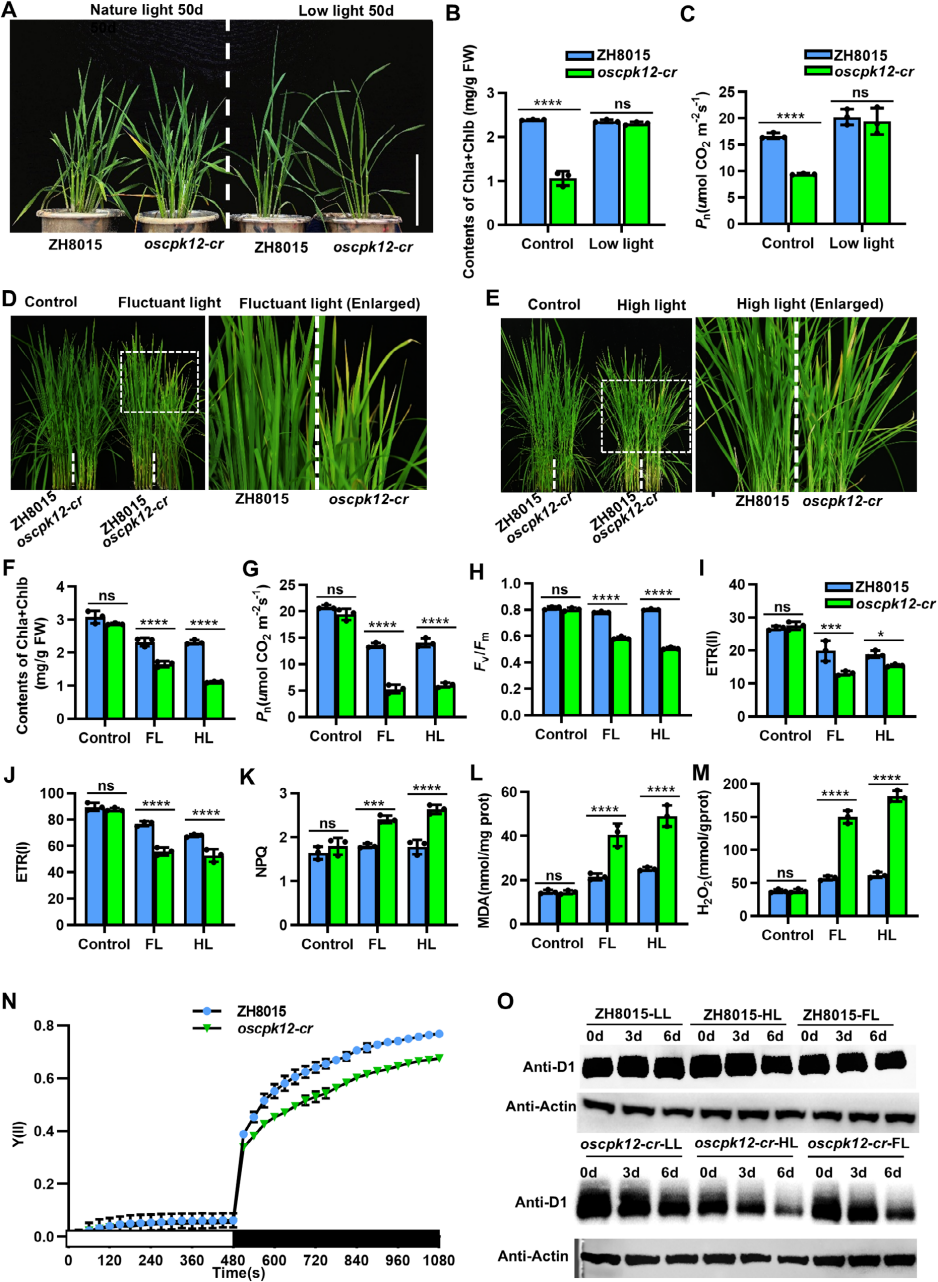
OsCPK12 may be involved in photosynthetic acclimation to light stress in rice. (A) Phenotypic differences between ZH8015 and *oscpk12*‐*cr* were observed under control and low‐light (LL) conditions at approximately 50 dps. (B) Chlorophyll, including chla and chlb, was quantified in the leaves of ZH8015 and *oscpk12*‐*cr* plants under control and LL conditions at 50 dps (n = 3 biological replicates). (C) Net photosynthetic rate (*P*_n_) was quantified in the leaves of ZH8015 and *oscpk12*‐*cr* under both control and LL conditions at 50 dps (n = 3 biological replicates). (D) Phenotypic variations between ZH8015 and *oscpk12*‐*cr* were noted after exposure to control and fluctuating‐light (FL) conditions for five days. (E) Phenotypic variations between ZH8015 and *oscpk12*‐*cr* were noted after exposure to control and high‐light (HL) conditions for three days. (F–M) Physiological parameters of ZH8015 and *oscpk12*‐*cr* under control, FL, and HL conditions: chlorophyll a + b content (F), *P*_n_ (G), maximum photochemical efficiency of PSII (*F*_v_/*F*_m_; H), electron transport rate of PSII (ETR(II); I), electron transport rate of PSI (ETR(I); J), non‐photochemical quenching (NPQ; K), malondialdehyde (MDA) content (L), and hydrogen peroxide (H_2_O_2_) content (M) (n = 3 biological replicates). (N) Dark‐light transition (480s) and relaxation (600s) of Y(II) in ZH8015 and *oscpk12*‐*cr* plants during the dark‐to‐light transition (black bar indicates light exposure). (n = three biological replicates). Data represent mean ± SD. (O) Immunoblot analysis of D1 protein levels in ZH8015 and *oscpk12*‐*cr* under LL, HL, and FL conditions at 0, 3, and 6 days. Actin was used as a loading control. For (B–C and F–M), data are given as mean ± SD. Two‐way ANOVA, followed by Fisher's least significant difference (LSD) post‐hoc test, was used to determine significance. Statistical significance was determined using a threshold of *p* < 0.05, with asterisks in the figures indicating varying significance levels: * (*p* < 0.05), *** (*p* < 0.001), and **** (*p* < 0.0001).

Previously, we identified a rice leaf senescence mutant *oscpk12‐cr* that affects chlorophyll synthesis, chloroplast development, and photosynthesis [25]. In this study, we found that light induces *OsCPK12* expression*. OsCPK12* overexpression also enhances the chlorophyll content and net photosynthetic rate (*P*_n_) in rice [25]. This suggests that OsCPK12 plays a role in regulating photosynthesis. To investigate whether the premature senescence phenotype of the *oscpk12‐cr* mutant is light‐dependent, we exposed ZH8015 and *oscpk12*‐*cr* to various lighting conditions (Figure S1). Consistently, the *oscpk12*‐*cr* mutant displayed premature senescence (Figure 1A) and significantly lower chlorophyll levels than ZH8015 after 50 days under natural light (Figure 1B,C). Notably, the premature senescence phenotype of *oscpk12*‐*cr* was entirely suppressed under low light conditions but reappeared within five days after returning to normal light (Figure 1A, Figure S2). We further confirmed that *OsCPK12* expression was induced by light (Figure S3), indicating that light triggered premature senescence in *oscpk12*‐*cr*. To validate this, we exposed ZH8015 and *oscpk12*‐*cr* to FL (cycles of 4 min of low light (100 *µm*ol photons m^−2^ s^−1^) and 1 min of high light (1200 *µm*ol photons m^−2^ s^−1^) and high light (900 *µm*ol photons m^−2^ s^−1^). As expected, *oscpk12*‐*cr* cells showed premature senescence after five days of fluctuating (Figure 1D) and high (Figure 1E) light. Additionally, the chlorophyll levels, *P*_n_, maximum quantum yield of PSII photochemistry (*F*_v_/*F*_m_), electron transport rate of PSII (ETR(II)), and electron transport rate of PSI (ETR(I)) in *oscpk12*‐*cr* were notably lower than those in ZH8015 (Figure 1F–J). In contrast, NPQ was markedly increased in *oscpk12*‐*cr* cells (Figure 1K). Simultaneously, hydrogen peroxide (H_2_O_2_) and malondialdehyde (MDA) levels were significantly elevated (Figure 1L,M). Consistent with these photosynthetic differences, dark relaxation analysis of Y(II) further revealed that the Y(II) value of *oscpk12*‐*cr* recovered to approximately 0.675 following light‐off conditions, whereas that of the WT recovered to 0.768 (Figure 1N). In addition, rapid degradation of the D1 protein was observed in *oscpk12*‐*cr*, the core component of the PSII reaction center (Figure 1O). These results suggest that high light‐induced oxidative stress and impaired PSII repair in *oscpk12*‐*cr* and *OsCPK12*may contribute to photosynthetic acclimation to light stress.

### 2.2 OsCPK12 Interacts With and Phosphorylates OsAtpD1 at Ser8

**FIGURE 2:**
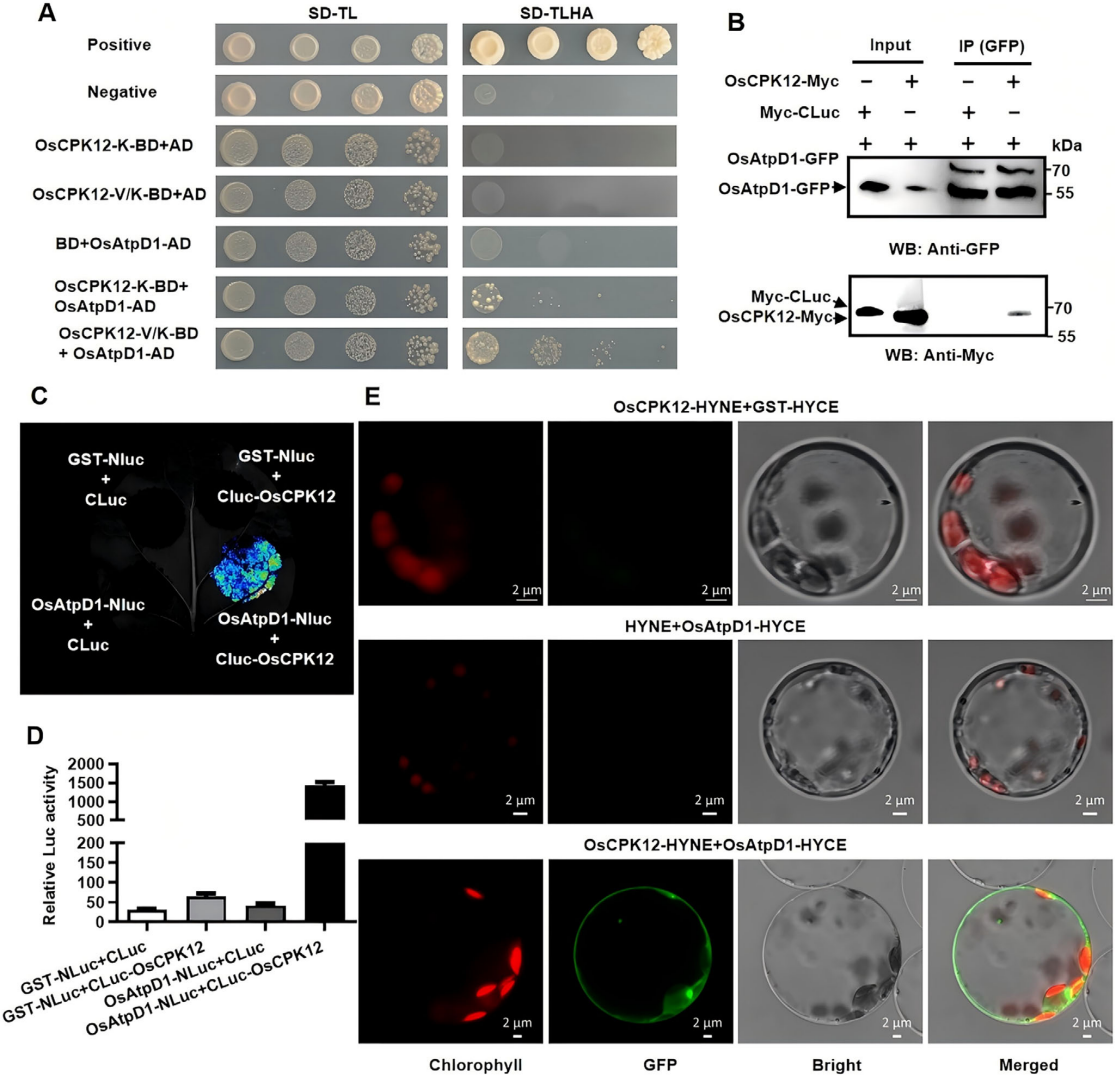
Interaction between OsCPK12 and OsAtpD1. (A) Yeast two‐hybrid (Y2H) assays were conducted to assess the interaction between OsCPK12 and OsAtpD1. Yeast cells expressing pGBKT7‐p53 and pGADT7‐T served as positive controls, whereas cells expressing pGBKT7‐Lam and pGADT7‐T served as negative controls. SD‐LW represents SD‐Leu/Trp, and SD‐LWHA stands for SD‐Leu/Trp/His/Ade. (B) Co‐immunoprecipitation (Co‐IP) assays were performed to investigate the interaction between OsCPK12 and OsAtpD1. Total protein was extracted from the rice protoplasts transfected with OsCPK12‐Myc/Myc‐Cluc or OsAtpD1‐GFP. Immunoprecipitation was performed using anti‐GFP sepharose beads. Both the crude lysate and the immunoprecipitated proteins were probed using anti‐GFP and anti‐Myc antibodies. (C–D) The interaction between OsCPK12 and OsAtpD1 was confirmed using the LUC complementation imaging (LCI) assay (C). OsCPK12‐CLuc and NLuc‐OsAtpD1 co‐infiltrate into *Nicotiana benthamiana*. GST‐Nluc, and‐CLuc were used as negative controls. (C) Luminescence signals were captured using a low‐light, cooled CCD imaging system two days post‐infiltration. (D) Quantitative analysis of LUC activity in leaves is shown in (C) (n = 3 biological replicates). Data represent mean ± SD. (E) Bimolecular fluorescence complementation (BiFC) assays showing that OsCPK12 interacts with OsAtpD1. OsCPK12‐HYNE and OsAtpD1‐HYCE were transiently expressed in the rice protoplasts. After 12–24 h of transfection, fluorescence was observed using a laser scanning confocal microscope (ZEISS 700). OsCPK12‐HYNE /GST‐HYCE and HYNE, and OsAtpD1‐HYCE were used as the negative controls. Scale bars = 2 *µm*.

OsCPK12 consists of an N‐terminal variable (V) domain, a protein kinase (K) domain, an autoinhibitory junction (J) region, and a CLD with four EF‐hand motifs [24, 26, 27]. To explore the mechanism by which OsCPK12 regulates photosynthesis in rice, we conducted a yeast two‐hybrid (Y2H) screen. Truncated OsCPK12 variants containing either the K domain or both the V and K domains were fused to the Gal4DNA binding domain (BD) in a bait vector. This generated cells containing OsCPK12‐K‐BD and OsCPK12‐V/K‐BD, respectively. Our Y2H results showed that OsCPK12 interacts with the δ‐subunit of the chloroplast ATP synthase, which we named OsAtpD1 (Figure 2A). We confirmed this interaction using in vivo coimmunoprecipitation (Co‐IP), luciferase complementation imaging (LCI), and bimolecular fluorescence complementation (BiFC) assays. The OsCPK12‐Myc fusion protein was co‐immunoprecipitated with OsAtpD1‐GFP, as shown in Figure 2B. Additionally, co‐expression of OsAtpD1‐NLuc and CLuc‐OsCPK12 in *Nicotiana benthamiana* leaves yielded strong fluorescence. In contrast, the control group showed only the baseline LUC activity (Figure 2C,D). The BiFC assay revealed a strong GFP signal in the protoplasts of ZH8015 cells co‐expressing OsCPK12‐HYNE and OsAtpD1‐HYCE. No signal was detected in the cells co‐expressing OsCPK12‐HYNE or GST‐HYCE (Figure 2E). This confirms the specific interaction between OsCPK12 and OsAtpD1. Collectively, these results demonstrated that OsCPK12 interacts with OsAtpD1.

**FIGURE 3:**
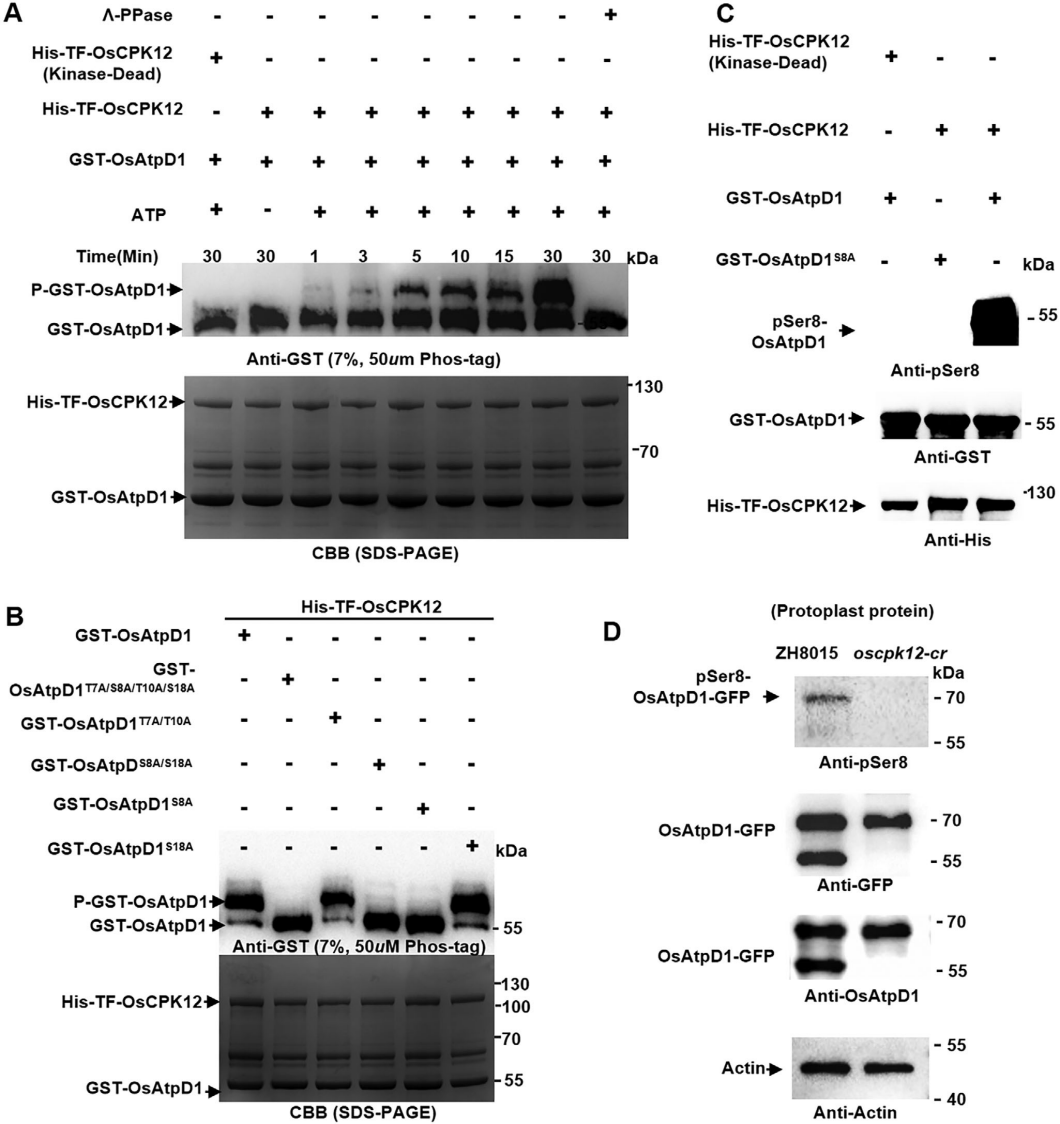
Phosphorylation of OsAtpD1 by OsCPK12 at Ser8. (A) OsCPK12 phosphorylates OsAtpD1 in vitro. The input proteins His‐TF‐OsCPK12 and GST‐OsAtpD1 were visualized by Coomassie Brilliant Blue (CBB) staining. Phosphorylation activity was evaluated through immunoblot analysis using 7% 50 *µ*M Phos‐Tag SDS‐PAGE. (B) OsCPK12 phosphorylated OsAtpD1 at Ser8. Protein phosphorylation was detected by immunoblotting with anti‐GST following Phos‐Tag SDS‐PAGE (top), whereas input protein loading was determined by CBB staining on a conventional SDS‐PAGE gel (bottom). pOsAtpD1 is diminished after treatment with lambda phosphatase (λ‐PPase), which is commonly used to dephosphorylate substrates. (C) Specificity of anti‐pSer8 antibody. Peptides of OsAtpD1 5–15 (pSer8) were spotted onto nitrocellulose membranes. Immunoblot analyses were performed using anti‐pSer8, anti‐GST, and anti‐His antibodies. (D) The OsAtpD1‐GFP fusion protein was transiently transformed into ZH8015 and *oscpk12*‐*cr* protoplasts. After 14 h, total protoplast proteins were extracted for Western blot (WB) detection using anti‐pSer8 and anti‐OsAtpD1 antibodies.

To determine whether OsAtpD1 was a substrate of OsCPK12, we performed an in vitro phosphorylation assay. Two bands were observed on the Phos‐Tag immunoblots, with the higher‐mobility band becoming more pronounced as the reaction time was extended. This indicated that GST‐OsAtpD1 was phosphorylated in vitro in the presence of OsCPK12. Moreover, phosphorylation of GST‐OsAtpD1 was eliminated by lambda phosphatase treatment (Figure 3A). These findings confirmed that OsAtpD1 is a substrate of OsCPK12.

To identify the phosphorylation sites in OsAtpD1, we excised the gel bands containing GST‐OsAtpD1. The GST‐OsAtpD1 were incubated with His‐TF‐OsCPK12 or His‐TF. The samples were analyzed by liquid chromatography‐tandem mass spectrometry (LC‐MS/MS). Four phosphorylation sites (Thr7, Ser8, Thr10, and Ser18) were identified in the His‐TF‐OsCPK12‐treated GST‐OsAtpD1, whereas they were absent in the His‐TF‐treated GST‐OsAtpD1 (Table S1). To validate these sites, we substituted the Ser (S) and Thr (T) residues with Ala (A) to mimic non‐phosphorylated residues. GST‐OsAtpD1S^T7A/T10A^ and GST‐OsAtpD1^S18A^ exhibited no significant effect on OsCPK12‐mediated phosphorylation compared to WT GST‐OsAtpD1 (Figure 3B). In contrast, GST‐OsAtpD1^S8A/T7A/T10A/S18A^, GST‐OsAtpD1^S8A/S18A^, and GST‐OsAtpD1^S8A^ showed minimal phosphorylation (Figure 3B). Therefore, it was inferred that Ser8 is the primary phosphorylation site of OsAtpD1 by OsCPK12. Further, antibodies were generated against the phospho‐Ser8 (pSer8) peptide to confirm this phosphorylation event. GST‐OsAtpD1 and GST‐OsAtpD1^S8A^ were incubated with His‐TF‐OsCPK12 or His‐TF‐OsCPK12 (kinase‐deficient). The phosphorylation at Ser8 was identified only in the GST‐OsAtpD1 protein incubated with His‐TF‐OsCPK12 (Figure 3C). Conversely, no phosphorylation was detected at Ser8 in the GST‐OsAtpD1 protein incubated with His‐TF‐OsCPK12 (kinase‐dead) or GST‐OsAtpD1^S8A^ incubated with His‐TF‐OsCPK12 (Figure 3C). To further verify the phosphorylation in OsAtpD1 at Ser8 by OsCPK12, we transiently transformed OsAtpD1‐GFP into ZH8015 and *oscpk12*‐*cr* protoplasts. Total protein was extracted after 14 h. Western blot (WB) detection was performed using anti‐pSer8, anti‐GFP, and anti‐OsAtpD1 antibodies. Consequently, Ser8‐phosphorylated OsAtpD1 was identified in ZH8015 cells (Figure 3D). Notably, anti‐GFP and anti‐OsAtpD1 antibodies detected two bands in the ZH8015 background but only one band in the *oscpk12*‐*cr* background (Figure 3D). We speculate that phosphorylation at Ser8 affects OsAtpD1 cleavage. These results indicated that OsCPK12 primarily mediates the phosphorylation of OsAtpD1 at Ser8.

### 2.3 The Phosphorylation Status of OsAtpD1 at Ser8 Promotes Its Chloroplast Translocation

**FIGURE 4:**
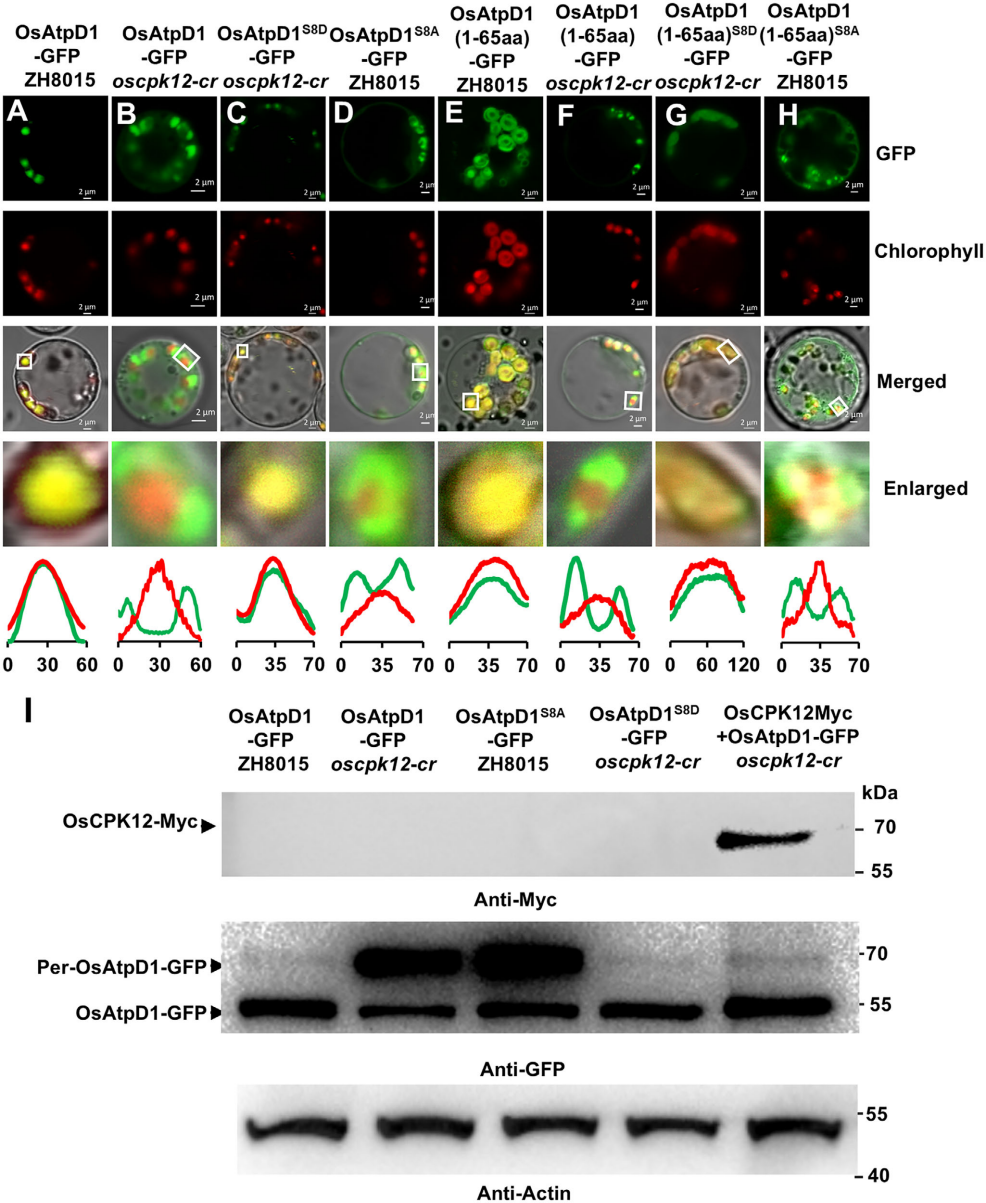
Subcellular localization and WB detection of OsAtpD1 and its variants in rice protoplasts of ZH8015 or *oscpk12*‐*cr*. (A) Localization of OsAtpD1 in ZH8015 rice protoplasts. The OsAtpD1‐GFP fusion protein was transiently transformed into the ZH8015 protoplasts. (B) Localization of OsAtpD1 in *oscpk12*‐*cr* protoplasts. The OsAtpD1‐GFP fusion protein was transiently transformed into the *oscpk12*‐*cr* protoplasts. (C) Localization of OsAtpD1^S8D^ in *oscpk12*‐*cr* protoplasts. The OsAtpD1^S8D^‐GFP fusion protein was transiently transformed into the *oscpk12*‐*cr* protoplasts. (D) Localization of OsAtpD1^S8A^ in ZH8015 protoplasts. The OsAtpD1^S8A^‐GFP fusion protein was transiently transformed into the ZH8015 protoplasts. (E) Localization of OsAtpD1(1‐63aa) in the ZH8015 protoplasts. The OsAtpD1(1‐63aa)‐GFP fusion protein was transiently transformed into the ZH8015 protoplasts. (F) Localization of OsAtpD1(1‐63aa) in *oscpk12*‐*cr* protoplasts. The OsAtpD1(1‐63aa)‐GFP fusion protein was transiently transformed into the *oscpk12*‐*cr* protoplasts. (G) Localization of OsAtpD1(1‐63aa)^S8D^ in *oscpk12*‐*cr* protoplasts. The OsAtpD1(1‐63aa)^S8D^‐GFP fusion protein was transiently transformed into the *oscpk12*‐*cr* protoplasts. (H) Localization of OsAtpD1(1‐63aa)^S8A^ in ZH8015 protoplasts. The OsAtpD1(1‐63aa)^S8A^‐GFP fusion protein was transiently transformed into the ZH8015 protoplasts. (A–H) The GFP and RFP fluorescence intensity profiles are shown in the box on the right. (I) WB detection of OsAtpD1 and its variants in ZH8015 or *oscpk12*‐*cr* rice protoplasts. OsAtpD1‐GFP and OsAtpD1^S8A^‐GFP were transformed into the ZH8015 protoplasts. OsAtpD1‐GFP, OsAtpD1^S8D^‐GFP, and OsCPK12‐Myc/OsAtpD1‐GFP were transiently transformed into the *oscpk12*‐*cr* protoplasts. After 14 h, the total protoplast protein was extracted for WB with anti‐GFP, anti‐Myc, and anti‐actin antibodies.

Nuclear genes encoding chloroplast proteins are translated into the cytoplasm. They are then imported to specific locations via transmembrane transporter complexes to perform specific functions. The chloroplast transit peptide (Ctp) is a pre‐protein sequence required for chloroplast targeting [28]. Results from the TargetP‐2.0 website and the ChloroP 1.1 server suggested that amino acids 1–54 of OsAtpD1 function as chloroplast transit peptides (Figure S4A,B). To investigate whether the phosphorylation at Ser8 influences OsAtpD1 chloroplast translocation, we performed a subcellular assay. Subcellular localization analysis revealed a GFP signal from OsAtpD1‐GFP in the chloroplasts of ZH8015 protoplasts (Figure 4A, Figure S5A). In contrast, this signal was predominantly observed in the cytoplasm of the *oscpk12*‐*cr* protoplasts (Figure 4B, Figure S5B,C). This indicates impaired transport of OsAtpD1‐GFP in *oscpk12*‐*cr*. Next, we fused mimic phosphorylated OsAtpD1^S8D^ and the Ctp of OsAtpD1(1‐65aa)^S8D^ to the N‐terminus of GFP. We also fused mimic non‐phosphorylated OsAtpD1^S8A^ and the Ctp of OsAtpD1(1‐65aa)^S8A^ to the N‐terminus of GFP. These constructs were transiently expressed in rice protoplasts derived from ZH8015 and *oscpk12*‐*cr*. The GFP signal from OsAtpD1^S8D^ was observed in the chloroplasts of *oscpk12*‐*cr* protoplasts, indicating the restoration of OsAtpD1 subcellular localization (Figure 4C). Conversely, in the ZH8015 background, the GFP signal from OsAtpD1^S8A^ was predominantly localized in the cytoplasm (Figure 4D), which was consistent with the subcellular localization of OsAtpD1 in *oscpk12*‐*cr*. The GFP signal from the truncated OsAtpD1(1‐65aa), which included only the chloroplast‐targeting signal peptide, mirrored the results obtained with the full‐length OsAtpD1 protein (Figure 4E–H). Therefore, combined with the results shown in Figure 3D, our study confirmed that OsCPK12 phosphorylates Ser8 on the Ctp of OsAtpD1. This phosphorylation facilitates the import of OsAtpD1 into chloroplasts, where it participates in ATP synthase assembly after cleavage. The OsAtpD1 precursor is synthesized in the cytosol. After being transported into the chloroplasts, it is cleaved to form functionally mature OsAtpD1.

To further verify whether the OsCPK12‐mediated phosphorylation of OsAtpD1 regulates its import efficiency into chloroplasts, we extracted total protein and examined the abundance of its precursor and mature forms. We found that OsAtpD1 exists predominantly as mature proteins in a WT background. In contrast, the precursor form was predominant in the *oscpk12‐cr* mutants. OsAtpD1^S8D^ was largely converted to mature OsAtpD1 in the *oscpk12*‐*cr* background. Consistently, the co‐expression of OsCPK12 and OsAtpD1 also led to the predominant accumulation of mature OsAtpD1 in the *oscpk12*‐*cr* background (Figure 4I). We also performed BiFC assays by co‐expressing OsCPK12‐HYNE with OsAtpD1^S8A^‐HYCE or OsAtpD1^S8D^‐HYCE in rice protoplasts. The fluorescence signals indicated that OsAtpD1^S8A^ and OsAtpD1^S8D^ interacted with OsCPK12 in the cytoplasm (Figure S6), demonstrating that phosphorylation at Ser8 had no influence on the interaction between OsAtpD1 and OsCPK12. Collectively, these results demonstrated that the OsCPK12‐mediated phosphorylation of OsAtpD1 at Ser8 is associated with the efficient accumulation of OsAtpD1 in chloroplasts. This process may further influence ATP synthase assembly and activity as well as rice photosynthesis.

### 2.4 The Phosphorylation Status of OsAtpD1 at Ser8 Is Necessary for Normal Photosynthesis

**FIGURE 5:**
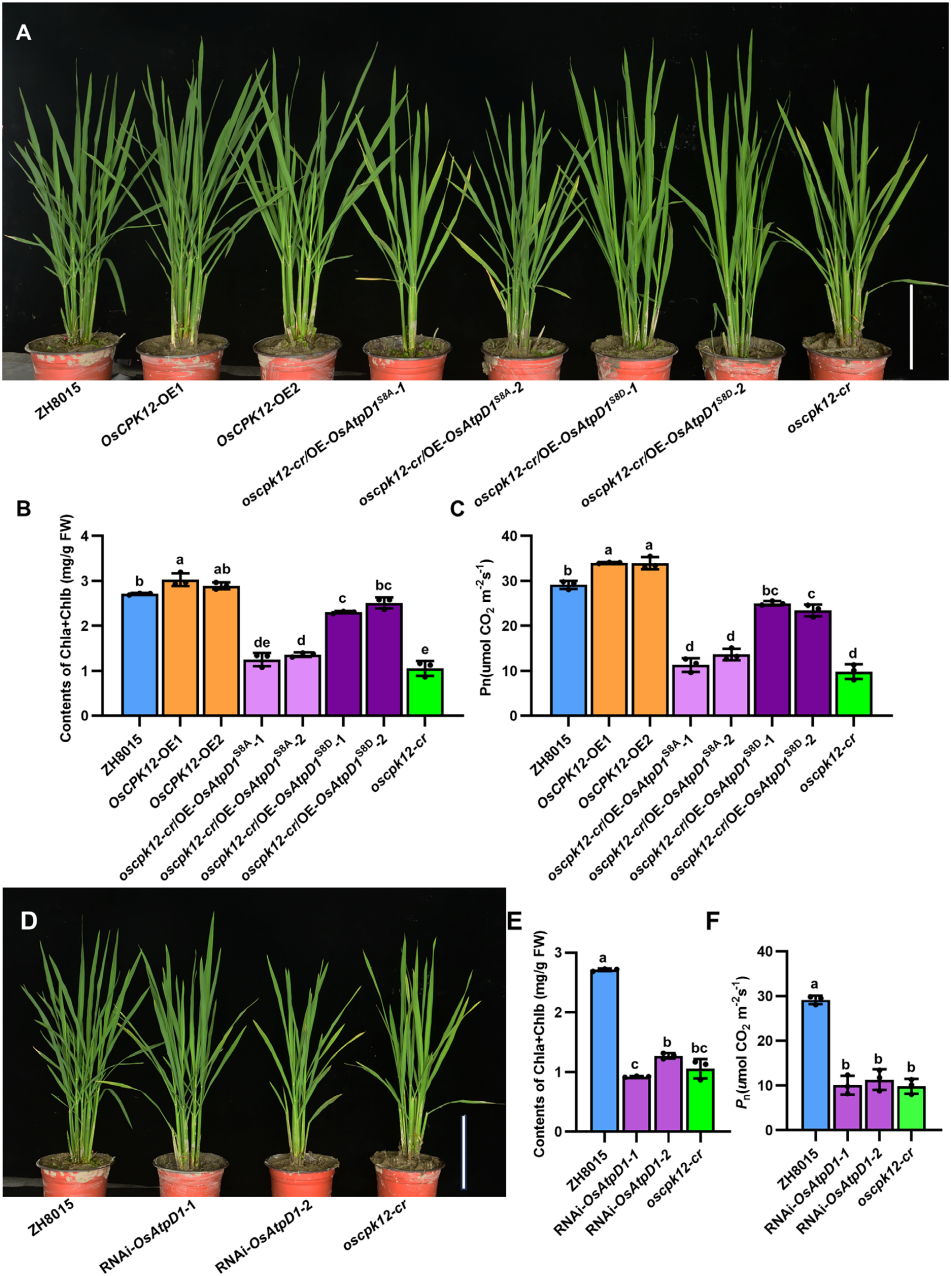
Phenotypic comparison, Chla + Chlb contents, and *P*_n_ of ZH8015, *oscpk12*‐*cr*, *oscpk12*‐*cr*/OE‐*OsAtpD1^S8A^*, *oscpk12*‐*cr*/OE‐*OsAtpD1^S8D^*, and RNAi‐*OsAtpD1*. (A) Phenotypic comparison of ZH8015, *oscpk12*‐*cr*, *oscpk12*‐*cr*/OE‐*OsAtpD1^S8A^*, and *oscpk12*‐*cr*/OE‐*OsAtpD1^S8D^*. Phenotypic images display only one representative line for each genotype. Scale Bar = 20 cm. (B, C) Chla + Chlb contents (B) and *P*_n_ (C) in the leaves of the ZH8015, *oscpk12*‐*cr*, *oscpk12*‐*cr*/OE‐*OsAtpD1^S8A^*, and *oscpk12*‐*cr*/OE‐*OsAtpD1^S8D^* lines (*n* = 3 biological replicates). (D) Phenotypic comparison of the ZH8015, *oscpk12*‐*cr*, and RNAi‐*OsAtpD1* lines. Scale Bar = 20 cm. (E, F) Chla + Chlb content (E) and *P*_n_ (F) in the leaves of ZH8015, *oscpk12*‐*cr*, and RNAi‐*OsAtpD1* lines (*n* = 3 biological replicates). For (B, C) and (E, F), the values are the mean ± SD. Different letters indicate significant differences as determined by one‐way ANOVA followed by Tukey's HSD post‐hoc test (*p* < 0.05).

To demonstrate that the photosynthetic impairment in *oscpk12*‐*cr* stems from the alteration of the phosphorylated Ser8 of OsAtpD1, we overexpressed both *OsAtpD1^S8A^* and *OsAtpD1^S8D^* in the *oscpk12*‐*cr* mutant. We identified two lines each of *oscpk12*‐*cr*/OE‐*OsAtpD1^S8A^* and *oscpk12*‐*cr*/OE‐*OsAtpD1^S8D^* (Figure S7). As expected, the *oscpk12*‐*cr*/OE‐*OsAtpD1^S8A^* lines showed phenotypes similar to *oscpk12*‐*cr*. In contrast, the *oscpk12*‐*cr*/OE‐*OsAtpD1^S8D^* lines displayed a milder premature senescent phenotype than both *oscpk12*‐*cr* and *oscpk12*‐*cr*/OE‐*OsAtpD1^S8A^* (Figure 5A). The chlorophyll contents and *P*_n_ in *oscpk12*‐*cr*/OE‐*OsAtpD1^S8D^* leaves were significantly higher than those in *oscpk12*‐*cr* and *oscpk12*‐*cr*/OE‐*OsAtpD1^S8A^*. However, these levels were slightly lower than those in ZH8015 (Figure 5B,C). Thus, *OsAtpD1^S8D^* overexpression in *oscpk12*‐*cr* lines nearly restored the senescent phenotype observed in *oscpk12*‐*cr*.

*OsAtpD1* encodes the δ‐subunit of chloroplast ATP synthase. This enzyme produces the most ATP in cells [29, 30]. To ascertain *OsAtpD1*’s significance in rice development, we generated *osatpd1*‐*cr* lines. We knocked out *OsAtpD1* in the ZH8015 background and identified two *osatpd1*‐*cr* lines (Figure S8A,B). Homozygous *osatpd1*‐*cr* lines showed severely stunted growth. These plants died approximately seven days after being transplanted into the soil (Figure S8A). Additionally, thylakoid protein levels (Lhca2, Lhca3, Lhca4, PsbO, Lhcb1, Lhcb2, Lhcb6, AtpB, AtpC, and AtpD) were lower in *osatpd1*‐*cr* lines compared to ZH8015 (Figure S8C). All *osatpd1*‐*cr* lines exhibited characteristics similar to *oscpk12‐cr*. These changes included decreased chloroplast ATP synthase activity and reduced chlorophyll content (Figure S8D,E). Furthermore, we generated two transgenic lines with RNAi targeting *OsAtpD1* (RNAi‐*OsAtpD1*) (Figure S7). The RNAi‐*OsAtpD1* plants displayed a leaf‐yellowing phenotype (Figure 5D). They also exhibited lower chlorophyll content and *P*_n_, similar to *oscpk12‐cr* (Figure 5E,F). In conclusion, *OsAtpD1* plays a crucial role in photosynthesis and overall rice growth.

**FIGURE 6:**
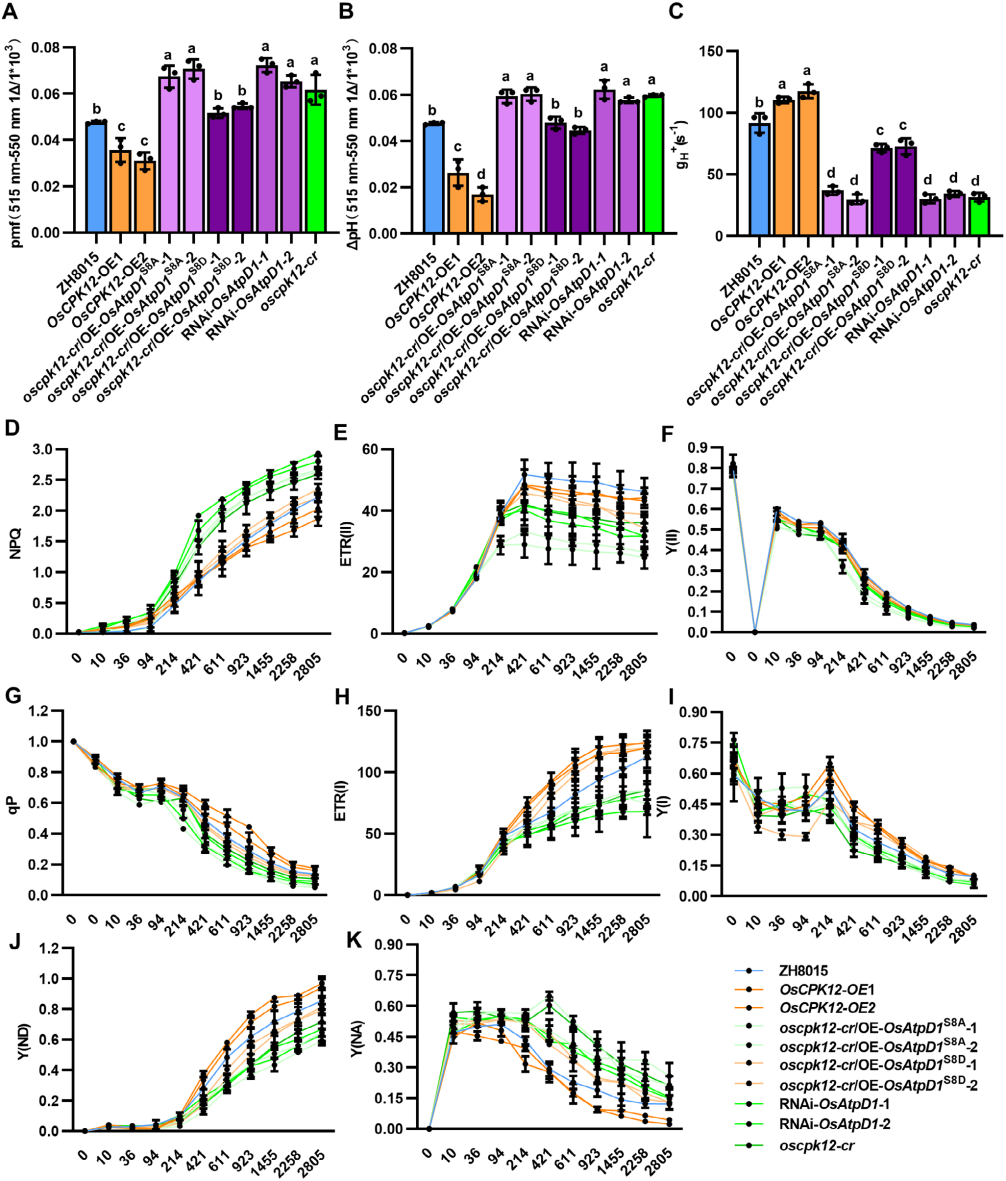
Characterization of ZH8015, *OsCPK12*‐OE, *oscpk12*‐*cr*/OE‐*OsAtpD1^S8A^*, *oscpk12*‐*cr*/OE‐*OsAtpD1^S8D^*, RNAi‐*OsAtpD1*, and *oscpk12*‐*cr* lines. (A, B) The proton motive force (PMF) (A) and pH gradient component (ΔpH) (B) across the thylakoid membrane in the leaves of the ZH8015, *oscpk12*‐*cr*/OE‐*OsAtpD1S8A*, *oscpk12*‐*cr*/OE‐*OsAtpD1S8D*, RNAi‐*OsAtpD1*, and *oscpk12*‐*cr* lines. These were determined by the slow relaxation phase of the electrochromic shift (ECS) signal after illuminating the leaves with red light at 843 *µ*mol photons m^−2^ s^−1^ for 10 min. (C) Fractional changes in proton conductivity across the thylakoid membrane (g_H_^+^) were estimated by the decay kinetics of the ECS, reflecting the activity of chloroplast ATP synthase. Values in A–C represent the mean ± SD (*n* = 3). Letters above the bars indicate significant differences (*p* < 0.05). *P*‐values were determined by one‐way analysis of variance (ANOVA), followed by Tukey's multiple comparison test. (D–K) Light intensity dependence of NPQ (D), ETR(II) (E), Y(II) (F), qP (G), ETR(I) (H), Y(I) (I), Y(ND) (J), and Y(NA) (K) was measured using an IMAGING‐PAM fluorometer with a series of light intensities (10, 36, 94, 214, 421, 611, 923, 1455, 2258, and 2805 *µ*mol photons m^−2^ s^−1^. All measurements are the averages of independent experiments with *n* = 3 biological replicates.

Further analyzing the phosphorylation of OsAtpD1 at Ser8 is crucial for understanding the electron transport chain in photosynthesis. We measured ATP synthase activity using fast dark‐relaxation kinetics of the maximum electrochromic absorption shift (ECS_T_), which quantifies the light‐induced PMF across the thylakoid membrane. The PMF is an important indicator of ATP synthase activity. We found that PMF was significantly higher in *oscpk12*‐*cr*, *oscpk12*‐*cr*/OE‐*OsAtpD1^S8A^*, and RNAi‐*OsAtpD1* than in the WT (Figure 6A). Further analysis revealed that this increase in PMF was primarily attributed to an increase in the transmembrane pH gradient (ΔpH), with no significant difference in Δψ (Figure 6B, Figure S8). This indicated higher proton accumulation in the thylakoid lumen of the *oscpk12*‐*cr*, *oscpk12*‐*cr*/OE‐*OsAtpD1^S8A^*, and RNAi‐*OsAtpD1* lines. We also found that the proton conductance (gH^+^) was consistently reduced to approximately 30%–40% of WT levels (Figure 6C). We analyzed the light‐intensity dependence of NPQ, ETR(II), and ETR(I), and found that the NPQ values of *oscpk12*‐*cr*, *oscpk12*‐*cr*/OE‐*OsAtpD1^S8A^*, and RNAi‐*OsAtpD1* were higher than those of the WT (Figure 6D). This may be explained by the enhanced lumen acidification resulting from the elevated ΔpH, which in turn triggers NPQ induction. Compared with the WT, lower ETR(II) levels and actual photochemical efficiency of PSII [Y(II)] were observed in *oscpk12*‐*cr*, *OsCPK12*‐OE, *oscpk12*‐*cr*/OE‐*OsAtpD1^S8A^*, *oscpk12*‐*cr*/OE‐*OsAtpD1^S8D^*, and RNAi‐*OsAtpD1* at light intensities exceeding 214 *µ*mol photons m^−2^ s^−1^ (Figure 6E,F). Photochemical quenching (qP) was elevated in *OsCPK12*‐OE and *oscpk12*‐*cr*/OE‐*OsAtpD1^S8D^* (Figure 6G). This suggests a higher proportion of open PSII reaction centers in *OsCPK12*‐OE and *oscpk12*‐*cr*/OE‐*OsAtpD1^S8D^* plants. Lower ETR(I) levels and actual photochemical efficiency of PSI (Y(I)) were observed in *oscpk12*‐*cr*, *oscpk12*‐*cr*/OE‐*OsAtpD1^S8A^*, and RNAi‐*OsAtpD1* at light intensities exceeding 421 *µ*mol photons m^−2^ s^−1^ (Figure 6H,I). This suggests LET rates were strongly suppressed in these three lines. PSI donor‐side limitation(Y(ND)) was higher in *OsCPK12*‐OE plants, indicating a more efficient electron supply to PSI (Figure 6J). In contrast, the PSI acceptor‐side limitation (Y(NA)) was higher in *oscpk12*‐*cr*, *oscpk12*‐*cr*/OE‐*OsAtpD1^S8A^*, and RNAi‐*OsAtpD1*, indicating blocked electron transfer at the PSI acceptor side (Figure 6K). This suggests that the OsCPK12‐mediated phosphorylation of OsAtpD1 at Ser8 is critical for maintaining proton‐gradient homeostasis across the thylakoid membrane. This homeostasis balances photochemical efficiency and photoprotection. In summary, our findings indicate that the phosphorylation of OsAtpD1 at Ser8 is crucial for photosynthesis. Specifically, it maintains electron transport, photosynthetic efficiency, and chloroplast ATP synthase activity. Moreover, *OsCPK12* overexpression confers greater tolerance to high light on rice.

### 2.5 Light Induces OsCPK12 Accumulation to Facilitate Chloroplast Translocation of OsAtpD1, Thereby Increasing the Photosynthetic Rate

**FIGURE 7:**
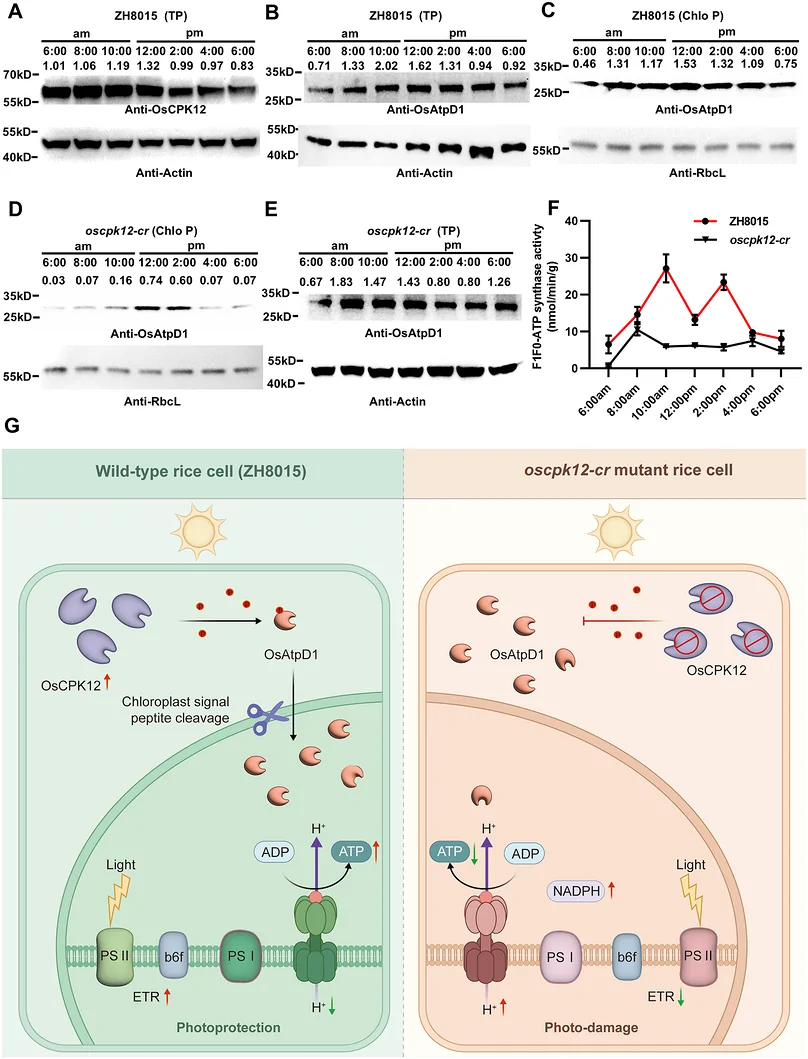
Light induces OsCPK12 phosphorylation in OsAtpD1 to facilitate chloroplast translocation. (A) The protein abundance of OsCPK12 in the total protein extracted from the leaves of ZH8015 plants at various time points throughout the day. (B) Protein abundance of OsAtpD1 in total protein extracted from the leaves of ZH8015 plants at different time points during the day. (C) Abundance of OsAtpD1 in the chloroplast proteins extracted from the leaves of ZH8015 plants at different times during the day. The anti‐RbcL antibody was used as a specific marker protein for chloroplasts to identify the chloroplast fractions and verify their subcellular localization. (D) Abundance of OsAtpD1 in the total protein extracted from the leaves of *oscpk12*‐*cr* plants at different time points during the day. (E) Abundance of OsAtpD1 in the chloroplast proteins extracted from the leaves of *oscpk12*‐*cr* plants at different times during the day. The anti‐RbcL antibody was used as a specific marker protein for chloroplasts to identify the chloroplast fractions and verify their subcellular localization. Band intensities were quantified using ImageJ software. Data in A–E are the relative levels of the target protein normalized to the corresponding actin band or RbcL band. (F) F1F0‐ATP synthase activity in the leaves of ZH8015 and *oscpk12*‐*cr* plants at various times during the day. Data represent mean ± SD (*n* = 3 biological replicates for each genotype). The experiments were repeated twice (A–E) or thrice (F) with similar results. (G) Light induces OsCPK12 to mediate phosphorylation of OsAtpD1 at Ser8. This facilitates its import into chloroplasts, aiding the assembly of the cpATPase complex. This assembly forms a catalytic motor that produces ATP. These processes are crucial for chloroplast development, chlorophyll synthesis, and photosynthesis in rice. In the *oscpk12*‐*cr* mutant, in which OsCPK12 function was lost, OsAtpD1 cannot be phosphorylated. As a result, non‐phosphorylated OsAtpD1 predominantly resided in the cytoplasm. This leads to a decrease in ATP synthase activity. Reduced ATP synthase activity lowers ATP production and causes an overaccumulation of protons in the thylakoid lumen, thereby inducing lumen overacidification and triggering NPQ. These changes further reduce ETR and photosynthetic efficiency and increase NADPH levels, ultimately resulting in leaf yellowing, premature senescence, and decreased grain yield.

Light is the primary energy source for plant photosynthesis [31]. Daily fluctuations in plant photosynthesis are closely associated with changes in light intensity. Our study showed that *OsCPK12* expression was induced by light. To investigate whether the OsCPK12‐OsAtpD1 pathway responded to diurnal light fluctuations, we examined *OsAtpD1* expression at various time points. *OsAtpD1* expression was also induced by light, peaking at 8 o'clock. This suggests that *OsAtpD1* was particularly sensitive to light (Figure S9). We further analyzed the abundance of OsCPK12 and OsAtpD1 proteins in total protein and chloroplast extracts from leaves of ZH8015 and *oscpk12‐cr* at different times during light shifts. Both OsCPK12 and OsAtpD1 protein levels increased in the total protein contents of ZH8015 and *oscpk12‐cr* over time. They peaked at 10 am (OsCPK12) and 12:00 pm (OsAtpD1) in ZH8015, and OsAtpD1 peaked at 8 am in *oscpk12‐cr* (Figure 7A,B,E). Among the chloroplast proteins of ZH8015, the OsAtpD1 protein abundance peaked at 12:00 pm (Figure 7C). A similar trend was observed for *oscpk12‐cr* chloroplast proteins, although at significantly lower levels (Figure 7D). However, OsAtpD1 in the total protein of *oscpk12‐cr/OE*‐*OsAtpD1^S8A^* increased over time, peaking at 2:00 pm. It was nearly undetectable in the chloroplast proteins of *oscpk12‐cr/OE*‐*OsAtpD1^S8A^* (Figure S10A,B). These findings suggest that increasing light intensity promotes the accumulation of OsCPK12 and OsAtpD1. Moreover, the OsCPK12‐mediated phosphorylation of OsAtpD1 is crucial for chloroplast translocation. Chloroplast ATP synthase activity in *oscpk12‐cr* was higher at 8:00 am and 4:00 pm, but was significantly lower than that in ZH8015 (Figure 7F). Interestingly, chloroplast ATP synthase activity in ZH8015 leaves was higher at 10:00 am and 2:00 pm. This may have resulted from the inhibition of photosynthesis at 12:00 pm (Figure 7F). The peak abundance of OsAtpD1 in ZH8015 chloroplasts at 12:00 pm may serve as a protective mechanism against light damage. Our results indicate that light induces OsCPK12 to phosphorylate OsAtpD1. This phosphorylation facilitates the chloroplast translocation of OsAtpD1 and contributes to photosynthetic acclimation to light stress. This mechanism is crucial for protecting rice from photodamage under HL conditions.

## 3 Discussion

### 3.1 OsCPK12 Phosphorylates OsAtpD1, Influencing Its Import into Chloroplasts

Chloroplast development is controlled by both the plastids and nuclei [32, 33]. In rice, more than 95% of the genes responsible for chloroplast development are encoded by the nucleus [34]. These nuclear‐encoded chloroplast proteins are synthesized in the cytoplasm and are then transported to specific chloroplast compartments via transmembrane translocon complexes to perform their functions. Nuclear genes encoding chloroplast proteins are often activated by light‐responsive transcription factors such as HY5 [35, 36, 37]. After transcription and translation in the cytosol, chloroplast preproteins undergo post‐translational modifications (PTMs). They are then imported into the chloroplast stroma through two translocon complexes: the translocon at the outer envelope membrane (TOC) and the translocon at the inner envelope membrane (TIC) [38]. Sequences that direct proteins to chloroplasts are known as Ctp. These Ctps are essential for targeting proteins to the chloroplasts [28]. Phosphorylation of Ctps plays a crucial role in regulating preprotein import [39, 40, 41]. Toc33/34 is a core component of the TOC complex and is indispensable for recognizing and binding nuclear‐encoded chloroplast pre‐proteins. Toc34 specifically interacts with the phosphorylated precursor protein preSSU [42]. STY kinase‐dependent transit peptide phosphorylation promotes 14‐3‐3‐Hsp70 complex formation, which enhances precursor targeting and high‐affinity docking to GTP‐bound Toc34. Phosphorylated precursors show stronger Toc34 binding than non‐phosphorylated forms, whereas defective phosphorylation impairs TOC recognition, suppresses chloroplast protein import, and disturbs chloroplast development [42, 43]. Our study shows that OsCPK12 interacts with OsAtpD1, the δ subunit of chloroplast ATP synthase, and phosphorylates Ser8 in Ctp. We found that the transport efficiency of OsAtpD1 was significantly reduced in *oscpk12*‐*cr* mutants (Figure 4). Therefore, phosphorylation of OsAtpD1 at Ser8 by OsCPK12 is associated with the efficient accumulation of OsAtpD1 in chloroplasts. In contrast, most of the green fluorescence of OsAtpD1‐GFP in *oscpk12*‐*cr* mutants was distributed around the chloroplasts. This indicates that non‐phosphorylated OsAtpD1 can still target the outer membrane of chloroplasts. Reduced import into chloroplasts may result from the lower recognition efficiency of the TOC complex. Collectively, these findings suggest that OsCPK12‐dependent phosphorylation of OsAtpD1 at Ser8 may promote OsAtpD1 import into chloroplasts, laying the foundation for its subsequent role in photosynthetic regulation.

### 3.2 The OsCPK12‐OsAtpD1 Module Regulates Photosynthesis, Photoprotection, and Acclimation in Rice

The OsCPK12‐OsAtpD1 molecular module is essential for regulating rice photosynthetic efficiency and photoprotection under HL stress, which induces excessive excitation pressure and photodamage to the photosystems, thereby limiting rice photosynthetic efficiency and growth. Our results identified the OsCPK12‐mediated phosphorylation of OsAtpD1 at Ser8 is associated with HL adaptation. The PMF provides energy for ATP synthesis and regulates multiple photoprotective mechanisms. Consistent with previous reports that reduced ATP synthase activity elevates PMF to trigger photoprotection [21], *oscpk12*‐*cr*, *oscpk12*‐*cr*/OE‐*OsAtpD1^S8A^*, and RNAi‐*OsAtpD1* showed increased PMF, ΔpH, and reduced gH^+^ in (Figure 6A–C). However, *oscpk12*‐*cr*/OE‐*OsAtpD1^S8D^* showed a decreased PMF and ΔpH than *oscpk12*‐*cr*. Therefore, loss of function or phosphorylation of OsAtpD1 at Ser8 impairs gH^+^, leading to lumen overacidification. Previous studies have demonstrated that PMF increases with increasing light intensity, activating diverse photoprotective responses, including induction of NPQ [12, 14, 15, 16]. Consistently, our study showed higher NPQ in *oscpk12*‐*cr*, *oscpk12*‐*cr*/OE‐OsAtpD1^S8A^, and RNAi‐*OsAtpD1* than in ZH8015, likely because lumen overacidification triggered NPQ for photoprotection (Figure 6D). These lines also exhibited lower photochemical quenching (qP), ETR, and PSI donor‐side limitation (Y(ND)), along with elevated PSI acceptor‐side limitation (Y(NA)), compared to *oscpk12*‐*cr*/OE‐*OsAtpD1^S8D^*, *OsCPK12*‐OE, and ZH8015 (Figure 6E–K). This confirms that the phosphorylation at Ser8 optimizes LET from PSII to PSI, ensuring a robust electron supply to PSI, whereas loss‐of‐function mutants show a PSI acceptor‐side bottleneck due to compromised ATP synthase activity. We also found that the ATP content was lower in the *oscpk12*‐*cr*, *oscpk12*‐*cr*/OE‐OsAtpD1^S8A^, and RNAi‐*OsAtpD1* than in *oscpk12*‐*cr*/OE‐*OsAtpD1^S8D^*, *OsCPK12*‐OE, and ZH8015; *oscpk12*‐*cr*/OE‐*OsAtpD1^S8D^* exhibited a moderately reduced ATP content relative to ZH8015, while maintaining higher ATP levels than *oscpk12*‐*cr*, *oscpk12*‐*cr*/OE‐OsAtpD1^S8A^, and RNAi‐*OsAtpD1* (Figure S12A). NADPH content was significantly elevated in the *oscpk12*‐*cr*, *oscpk12*‐*cr*/OE‐OsAtpD1^S8A^, and RNAi‐*OsAtpD1* compared to that in *oscpk12*‐*cr*/OE‐*OsAtpD1^S8D^*, *OsCPK12*‐OE, and ZH8015. Although *oscpk12*‐*cr*/OE‐*OsAtpD1^S8D^* had higher NADPH levels than ZH8015, its NADPH content was still lower than that of *oscpk12*‐*cr*, *oscpk12*‐*cr*/OE‐OsAtpD1^S8A^, and RNAi‐*OsAtpD1* lines (Figure S12B). Such changes are likely attributable to feedback inhibition of photosynthetic electron transport, dysregulation of the proton gradient, and impaired NADPH consumption in the Calvin cycle. Reduced ATP synthase activity induces lumen overacidification, triggering photosynthetic control that suppresses plastoquinol reoxidation by Cytb_6_f (a rate‐limiting step in LET), reducing ETR and exacerbating NADPH accumulation via preferential electron flow to NADPH generation over CEF‐mediated ATP production. These results further substantiate that phosphorylation at Ser8 is associated with efficient accumulation of OsAtpD1 in chloroplasts.

The D1 repair cycle continuously replenishes the damaged D1 proteins to maintain PSII function, mitigate photooxidative stress, and sustain normal plant photosynthesis. We also found that under HL conditions, D1 protein levels declined more rapidly in the *oscpk12*‐*cr* mutant (Figure 1O). OsCPK12 impairs plants' photosynthetic repair capacity. The phosphorylation of D1 is also closely related to the reversible phosphorylation of the chloroplast protein transport [44]. High light levels also induce the phosphorylation and dephosphorylation of D1, which plays a crucial role in the PSII repair cycle [45, 46]. Despite the photoprotective responses, *oscpk12*‐*cr* plants exhibited aggravated photodamage and premature senescence, which were suppressed under low light but induced under HL and FL (Figure 1A,D,E). The protein abundance of OsCPK12 and OsAtpD1 in WT plants increased with light intensity and peaked at noon (Figure 7A,B). However, ATP synthase activity peaked at 10:00 am and 2:00 pm, with a clear trough at noon (Figure 7F). This indicated that ATP synthase activity was not strictly linearly related to OsAtpD1 protein abundance. One possible explanation is the decoupling of protein levels from enzyme activity, a common regulatory phenomenon in biological systems. The uncoupling of non‐peaking OsAtpD1 and midday ATP synthase activity arises from the post‐translational and electrochemical regulation of chloroplast ATP synthase, and not subunit abundance [47]. Reversible γ‐subunit thiol redox gating and thylakoid hyperpolarization under midday light suppress synthase rotation regardless of subunit dosage, while PMF sensing at the rotor–stator interface further dissociates steady‐state subunit levels from real‐time catalytic capacity. We infer that noon OsAtpD1 accumulation compensates for persistently high light at the transcriptional and translational levels, yet redox‐ and voltage‐driven inhibition of the assembled complexes limits ATP production, jointly causing midday activity depression. Our previous study also revealed that OsCPK12‐mediated phosphorylation of OsCATA and OsCATC elevates catalase activity in rice [48]. Therefore, we hypothesized that the OsCPK12‐mediated phosphorylation of OsAtpD1, OsCATA, and OsCATC coordinately regulates plant responses to HL stress. Correspondingly, our previous study found that the expression levels of photosynthesis‐related genes and their encoded proteins were significantly decreased in *oscpk12*‐*cr* mutants (Figure S13). Y(II) in *oscpk12*‐*cr* recovered to approximately 0.675 after light‐off conditions, whereas that in ZH8015 recovered to 0.768 (Figure 1N), and degradation of the D1 protein was rapid in *oscpk12*‐*cr* under HL conditions (Figure 1O). We also found higher levels of H_2_O_2_ and MDA in *oscpk12*‐*cr* cells under HL conditions (Figure 1L,M). These results indicated abnormal photosynthetic damage repair in *oscpk12*‐*cr* mutants. Premature senescence is inevitable as damage accumulates. This may be the underlying molecular reason for the complete suppression of the early senescence phenotype of *oscpk12*‐*cr* mutants under low light, but its strong induction under HL, and FL.

Photosynthetic acclimation refers to rapid and reversible changes in the photosynthetic rate due to fluctuating environmental conditions. These include light intensity, temperature, CO_2_ concentration, and humidity [49]. Photosynthetic acclimation is the ability of plants to respond quickly to environmental changes. This mainly involves the coordinated regulation of light and dark reactions. OsAtpD1 expression in the WT chloroplasts was significantly and positively correlated with light intensity (Figure 7C). This indicates that OsAtpD1 responds passively to changes in light. Such a response helps to coordinate light and dark reactions and supports maximum photosynthetic efficiency. In the *oscpk12*‐*cr* mutant, OsAtpD1 exhibited a similar light response pattern. However, protein accumulation in the chloroplasts was significantly lower than that in the WT (Figure 7D). Consequently, ATP synthase activity and CO_2_ assimilation rate were both reduced compared to those of the WT (Figure 5C, Figure 6A–C). Based on these results, we concluded that OsAtpD1 is critical for the coordinated regulation of light and dark reactions. This regulation largely depended on OsCPK12‐mediated phosphorylation. Plants with reduced AtpD abundance exhibit a decreased linear electron flow (LEF) and increased NPQ [50, 51]. We also observed decreased LET, PMF, and CO_2_ assimilation rates in RNAi‐*OsAtpD1* plants, similar to *oscpk12*‐*cr* plants (Figures 5F and 6A–K). In contrast, overexpressing *AtpD* increased the abundance and activity of ATP synthase. This leads to a higher electron transport rate (J) and CO_2_ assimilation rate at high CO_2_ and irradiance, even with WT levels of other thylakoid complexes in rice [52]. In our study, we found that homozygous *osatpd1*‐*cr* knockout mutants exhibited seedling lethality (Figure S8A), a phenotype distinct from the premature senescence of *oscpk12*‐*cr*. Notably, the RNAi‐*OsAtpD1* transgenic lines recapitulated the characteristic defects of *oscpk12*‐*cr*. Whereas the *oscpk12*‐*cr* mutant only partially impaired *OsAtpD1* trafficking by eliminating phosphorylation, and *osatpd1*‐*cr* completely depleted the OsAtpD1 protein. Thus, we hypothesized that OsAtpD1 executes essential biological functions independent of OsCPK12‐dependent phosphorylation, exerting broad and irreplaceable effects throughout chloroplast biogenesis. This is strongly supported by Figure S8C, which shows near‐complete loss of thylakoid proteins in homozygous *osatpd1*‐*cr* seedlings. Specifically, we clarified that the seedling lethal phenotype of homozygous *osatpd1*‐*cr* resulted from the complete loss of the OsAtpD1 protein, which severely disrupted chloroplast biogenesis and thylakoid protein accumulation, thereby causing irreversible damage to the photosynthetic apparatus. In contrast, the *oscpk12*‐*cr* mutant impaired only the phosphorylation and chloroplast import of OsAtpD1, resulting in partial functional attenuation rather than complete protein depletion. Our results support the existence of essential OsAtpD1 functions that are uncoupled from OsCPK12‐dependent phosphorylation during chloroplast biogenesis.

OsCPK12 participates in multiple signaling pathways [53, 54]. Our previous studies demonstrated that OsCPK12 enhances catalase activity by phosphorylating OsCATA and OsCATC, thereby improving plant tolerance to oxidative stress [48]. Phosphomimetic OsAtpD1 failed to fully rescue the premature senescence phenotype of *oscpk12‐cr* mutants. Therefore, we inferred that the OsCPK12‐OsAtpD1 phosphorylation pathway only partially accounted for the photosynthetic dysfunction observed in *oscpk12*‐*cr*. Two plausible interpretations are presented. First, the OsCPK12‐OsAtpD1 phosphorylation pathway only partially accounts for the photosynthetic dysfunction observed in *oscpk12*‐*cr*. We propose that OsCPK12 confers photoprotection and suppresses leaf senescence via dual regulatory branches: one relies on OsAtpD1 phosphorylation for chloroplast function maintenance, and the other operates through phosphorylated OsCATA and OsCATC to alleviate oxidative damage [48]. Second, OsCPK12 mediates photoprotection and suppression of leaf senescence through multiple independent regulatory cascades. In addition to the three characterized substrates, OsAtpD1, OsCATA, and OsCATC, OsCPK12 likely targets additional uncharacterized proteins to cooperatively sustain photosynthetic homeostasis. Consistently, *OsCPK12* overexpression significantly increased ATP synthase activity, CO_2_ assimilation capacity, and grain yield (Figure S14), further highlighting its critical role in optimizing photosynthetic performance. Therefore, modulating *OsCPK12* expression is a promising strategy for breeding light‐stress‐tolerant rice varieties. Such genetic modifications enable plants to maintain stable photosynthetic efficiency under FL conditions, mitigate yield penalties caused by photoinhibition, and ultimately improve rice productivity.

Collectively, the OsCPK12‐OsAtpD1 module coordinately regulates photosynthetic acclimation, photoprotection, and photosynthetic repair by maintaining ATP synthase activity and proton‐gradient homeostasis, thereby providing a strategy to enhance rice photosynthetic resilience under light stress. As shown in Figure 7G, in ZH8015, light‐induced OsCPK12 phosphorylated OsAtpD1, facilitating its chloroplast import and participation in ATP synthase assembly. This optimizes the photosynthetic electron transport efficiency and ATP synthase activity. Ultimately, it modulates photosynthetic acclimation to cope with the FL conditions. In contrast, in the *oscpk12*‐*cr* mutant, in which OsCPK12 function was lost, OsAtpD1 cannot be phosphorylated. As a result, non‐phosphorylated OsAtpD1 predominantly resided in the cytoplasm. This leads to a decrease in ATP synthase activity. Reduced ATP synthase activity reduces ATP production and causes proton overaccumulation in the thylakoid lumen, thereby inducing lumen overacidification and triggering NPQ. These changes further reduce ETR and photosynthetic efficiency, and increase NADPH levels, ultimately resulting in leaf yellowing, premature senescence, and decreased grain yield. *OsCPK12* overexpression was sufficient to increase ATP synthase activity and CO_2_ assimilation rates. Our findings revealed the molecular mechanisms by which ATP synthase regulates photosynthetic acclimation. They also proposed a new strategy to improve photosynthetic performance.

## 4 Materials and Methods

### 4.1 Plant Materials and Growth Conditions

ZH8015 and *oscpk12‐cr* plants were cultivated under natural field conditions. In the low‐light treatment, plants were grown under natural sunlight with a single layer of black netting to reduce light intensity. Twenty days post‐sowing (dps), ZH8015 and *oscpk12‐cr* mutant plants were planted under field conditions as controls or in fields covered with black nets to simulate low‐light treatments. The third upper leaves of ZH8015 and *oscpk12‐cr* at the tillering stage (50 dps) were used for physiological and phenotypic measurements and for *OsCPK12* and *OsAtpD1*expression analyses. Both ZH8015 and *oscpk12‐cr* were cultured in an incubator under a diurnal photoperiod (14 h dark/10 h light) at a light intensity of 300 *µm*ol photons m^−2^ s^−1^, serving as the control. For HL and FL treatment, a portion of bothZH8015 and *oscpk12‐cr* plants (15 dps) were transferred to an incubator under a diurnal photoperiod (14 h dark/10 h light) with a light intensity of 900 *µm*ol photons m^−2^ s^−1^ as HL treatment and cycles of 4 min of low light (100 *µm*ol photons m^−2^ s^−1^) and 1 min of high light (1200 *µm*ol photons m^−2^ s^−1^) as FL treatment for 5 days. The second leaves from these plants at 20 dps were used to assess gene expression and to measure *P*_n_ and chlorophyll content.

### 4.2 Measurement of Chlorophyll Content, *P*_n_, and Enzymatic Activity

Chlorophyll content and *P*_n_ were measured as described by [25]. Leaves (third from the top) of the ZH8015, *OsCPK12*‐OE, *oscpk12*‐*cr*/OE‐*OsAtpD1^S8A^*, *oscpk12*‐*cr*/OE‐*OsAtpD1^S8D^*, RNAi‐*OsAtpD1*, and *oscpk12*‐*cr* lines were collected at 50 dps for the measurements. Chloroplast ATP synthase (F1F0‐ATP synthase) activity was assessed using fresh leaf tissues from the ZH8015, *OsCPK12*‐OE, *oscpk12*‐*cr*/OE‐*OsAtpD1^S8A^*, *oscpk12*‐*cr*/OE‐*OsAtpD1^S8D^*, RNAi‐*OsAtpD1*, and *oscpk12*‐*cr* lines using commercial assay kits from Suzhou Comin Biotechnology Co., Ltd. (#FHTE‐1‐Y), following the manufacturer's instructions. First, the chloroplasts were extracted. Leaf tissue (0.1 g) was weighed and ground into a homogenate using 1 mL of Reagent 1, after which it was centrifuged at 200 g for 1 min at 4°C. Then, the supernatant was transferred into a new centrifuge tube and centrifuged at 1500 g for 5 min at 4°C. The supernatant was discarded, and 0.5 mL of Reagent 1 was added to the precipitate to form a chloroplast suspension for determining chloroplast ATP synthase activity. Second, 50 *µL* of the chloroplast suspension, 10 *µL* of Reagent 2, and 40 *µL* of Reagent 3 were mixed to react for 30 min at 25°C. Then, 50 *µL* of Reagent 4 was added to stop the reaction. Finally, 30 *µL* of the supernatant was mixed with 170 *µL* of the phosphorus reagent (H_2_O: Reagent 5: Reagent 6: Reagent 7 = 2:1:1:1), and the mixture was allowed to stand for 10 min, and the absorbance was measured at 660 nm. The specific experimental steps and calculation methods were performed according to the manufacturer's instructions. The second upper leaves of *osatpd1*‐*cr* were used for physiological measurements because the *osatpd1*‐*cr* mutant is seedling lethal.

### 4.3 Determination of H_2_O_2_ and MDA Contents

The H_2_O_2_ and MDA contents were measured using commercial assay kits obtained from the Nanjing Jiancheng Bioengineering Research Institute (Nanjing, China). The second upper leaves of ZH8015 and the *oscpk12*‐*cr* mutant were sampled after 5 days of HL and FL treatment, respectively. All procedures were performed following the manufacturer's instructions. Phenotypic values were presented as the mean of three biological replicates.

### 4.4 Determination of ATP and NADPH Contents in Chloroplasts

Chloroplasts were isolated from the third upper leaves of the ZH8015 and *oscpk12*‐*cr* mutant plants at 50 dps. Rice chloroplasts were isolated via density gradient centrifugation, following the protocol of the Chloroplast Extraction Kit (PTE010, Coolaber). All experiments were performed at 4°C under dim light conditions. Leaf tissues were homogenized in pre‐chilled homogenization buffer supplemented with 0.1% BSA. The homogenate was filtered through Miracloth and sequentially centrifuged at 400 × *g* and 1300 × *g* to collect crude chloroplasts. Intact chloroplasts were purified by discontinuous Percoll gradient centrifugation (3750 × *g*, 15 min), pelleted at 1700 × *g* for washing, and resuspended for subsequent experiments. The ATP and NADPH contents in chloroplasts were determined using commercial kits (Cat. No. NMK0005 and NMW0902; Norminkoda, Wuhan, China). All procedures were performed following the manufacturer's instructions. Phenotypic values were presented as the mean of three biological replicates.

### 4.5 Gene Expression Analysis

The third upper leaves of the WT ZH8015 were collected at 50 dps, every 2 h from 6:00 am to 8:00 pm, under both control and low‐light conditions for expression analysis of the *OsCPK12* and *OsAtpD1* genes. Total RNA was extracted using the RNA Prep Pure Plant Kit (TIANGEN, Beijing, China). cDNA was synthesized from total RNA using the ReverTra Ace qPCR‐RT Master Mix (Toyobo, Japan). qRT‐PCR was conducted using SYBR premix Ex Taq II (Takara, Japan) on a Light Cycler 480 II (Roche, Sweden), following the manufacturer's guidelines. *UBQ10* was used as the reference gene. The primer pairs used for qPCR are listed in Table S2.

### 4.6 Generation of Knockdowns and Knockouts of the *OsAtpD1* Gene

The generation of *OsAtpD1* knockdowns and knockouts followed the method described by [55]. The sequences of all primers used for vector construction or material genotyping are listed in Table S2.

### 4.7 Subcellular Localization of OsAtpD1

The coding sequences (CDS) of *OsAtpD1*, *OsAtpD1^S8A^*, *OsAtpD1^S8D^*, *OsAtpD1(1‐65aa)^S8A^* and *OsAtpD (1‐65aa)^S8D^* were fused to the N‐terminus of pYBA‐1132‐GFP [56]. OsAtpD1‐GFP was colocalized with mCherry in the rice protoplast of *oscpk12*‐*cr*. These recombinant proteins were transiently expressed in the rice protoplasts of ZH8015 and *oscpk12*‐*cr*. Fluorescence was visualized in rice protoplasts using a laser scanning confocal microscope (ZEISS 700) between 14 and 24 h post‐transfection. The primers used to generate the constructs are listed in Table S2.

### 4.8 Yeast Two‐Hybrid (Y2H) Assays

For the Y2H assay, specific domains of *OsCPK12* and the full‐length coding sequence of *OsAtpD1* were amplified and inserted into pGBKT7 or pGADT7 vectors. These constructs were then transformed into the Y2Hgold yeast strain, according to the manufacturer's guidelines (Clontech). The primers used for the amplification are listed in Table S2.

### 4.9 Coimmunoprecipitation (Co‐IP) Assays

The full‐length CDS of *OsCPK12* and *OsAtpD1* were fused to sequences encoding Myc and GFP tags, respectively, under the control of the 35S promoter. Cluc‐Myc and OsCPK12‐Myc were co‐expressed with OsAtpD1‐GFP in protoplasts of ZH8015 [57]. Total protein extraction and Co‐IP assays were conducted as previously described [48]. Immunoblotting was performed using anti‐Myc (TransGen; HT101) or anti‐GFP (TransGen; HT801), followed by incubation with a secondary antibody (HuaAn; HA1006). The immunoblot signals were visualized using Super‐ECL (Coolaber, SL1350). The primers used are listed in Table S2.

### 4.10 Firefly Luciferase Complementation Imaging (LCI) Assay

The full‐length CDS of *OsAtpD1* and glutathione‐S‐transferase (GST) amplified were cloned into the pCAMBIA1300‐NLuc vector to generate OsAtpD1‐NLuc and GST‐NLuc vectors, respectively. The full‐length CDS of *OsCPK12* were cloned into the pCAMBIA1300‐CLuc vector to generate CLuc‐OsCPK12. OsAtpD1‐NLuc and empty vectors CLuc, GST‐NLuc, CLuc‐OsCPK12, OsAtpD1‐NLuc, and CLuc‐OsCPK12 were co‐transformed into *N. benthamiana* leaves. After 48 h of infiltration, a D‐luciferin potassium salt (Coolaber, 115114‐35‐9) solution was applied to the leaves, and luciferase activity was measured with a Promega GloMax 20/20 Luminometer. The primers used for the LCI assay are listed in Table S2.

### 4.11 BiFC Assay

The cDNAs of *OsCPK12*, *OsAtpD1*, *OsAtpD1^S8A^*, and *OsAtpD1^S8D^* were cloned into 35S‐SPYNE and 35S‐SPYCE to generate the OsCPK12‐HYNE and OsAtpD1‐HYCE vectors, respectively. The constructs were transiently expressed in ZH8015 rice protoplasts. Fluorescence was visualized using a laser‐scanning confocal microscope (ZEISS 700) after transient expression for 14 to 24 h. The primers used for the BiFC assay are listed in Table S2.

### 4.12 Phosphorylation Assays in Vitro

His‐ and GST‐fused recombinant proteins were expressed in the *E*. *coli* strain BL21(DE3) and purified using a His‐tag Protein Purification Kit (Cat no: P2226 Beyotime Biotechnology) and BeaverBeadsTM GSH (Cat no.70601‐100 Xi Yan Technology). Approximately 2 microgram (*µ*g) of kinase and 2 *µ*g of substrate proteins were mixed with 1x kinase buffer (100 millimolar (mM) Tris‐HCl [pH 8.0], 5 mM DTT, 5 mM EGTA, 5 mM MgCl_2_, 0.5 mM CaCl_2_, and 100 micromolar (*µ*M) ATP) in a 30 microliter (*µ*L) total volume. The reaction mixture was incubated at 30°C for 30 min. For alkaline phosphatase‐mediated dephosphorylation, 500 U of fast alkaline phosphatase (λ‐PP, Thermo Fisher, USA) were added to the above kinase reaction buffer. The mixture was gently vortexed and incubated at 30°C for 30 min to complete the dephosphorylation. Phosphorylated proteins were examined using a 7% 50 *µ*M Phos‐Tag acrylamide Gel (Phos‐tag AAL‐107; Wako) and anti‐GST antibody (TransGen; HT801). The primers used are listed in Table S2.

### 4.13 Protein Extraction and Immunoblotting Analysis

The pCold TF vector (Takara), a fusion cold‐shock expression vector that expresses a 48‐kDa trigger factor chaperone as a soluble tag, also contains a His‐tag sequence. Therefore, we named it the His‐TF vector. His‐TF vectors containing full‐length OsCPK12 CDSs (His‐TF‐OsCPK12) and GST fusion with full‐length OsAtpD1 CDSs were constructed for pull‐down and phosphorylation assays. Mutant isomers of GST‐OsAtpD1^T7A/S8A/T10A/S18A^, GST‐OsAtpD1^T7A/T10A^, GST‐OsAtpD1^S8A/S18A^, GST‐OsAtpD1^S8A^, and GST‐OsAtpD1^S18A^ were developed to validate specific phosphorylation sites. The recombinants were transformed into *Escherichia coli* BL21, and protein expression was induced using 0.1 mM isopropyl β‐D‐thiogalactoside at 15°C for 12 h. The cells were harvested and analyzed by Sodium Dodecyl Sulfate‐Polyacrylamide Gel Electrophoresis (SDS‐PAGE), and the GST‐ and His‐tagged proteins in the supernatant were purified using BeaverBeads GSH (catalog no. 70601‐100, Xi Yan Technology) and a His‐tag protein Purification Kit (catalog no. P2226, Beyotime Biotechnology, China). The protein concentration was assessed using an NCM BCA Protein Assay Kit (catalog no. WB6501; New Cell and Molecular Biotechnology). The primers used for the expressed protein constructs are listed in Table S2.

Total protein was extracted as previously described [48] and quantified to the same concentration using an NCM BCA Protein Assay Kit (catalog no. WB6501; New Cell and Molecular Biotechnology). Chloroplast proteins were extracted using a commercial Chloroplast Protein Isolation Kit (catalog No. BB‐3179, BestBio, Shanghai, China). Fresh leaf tissues from ZH8015, *oscpk12*‐*cr*, and *oscpk12*‐*cr*/OE‐OsAtpD1^S8A^ plants were collected for chloroplast protein isolation following the manufacturer's instructions. The *RbcL* gene is encoded by the chloroplast genome and encodes the large subunit of ribulose‐1,5‐bisphosphate carboxylase/oxygenase (RuBisCO, LSU). Accordingly, anti‐RbcL was selected as a chloroplast‐specific marker to evaluate the purity of the isolated chloroplast samples. Antibodies used for immunoblotting analysis of thylakoid membrane proteins were purchased from Agrisera (Vännäs, Sweden), including anti‐Lhca2 (AS01006), anti‐Lhca3 (AS01007), anti‐Lhca4 (AS01008), anti‐PsbO (AS05092), anti‐Lhcb1 (AS01004), anti‐Lhcb2 (AS01003), anti‐Lhcb6 (AS01010), anti‐AtpB (AS05085), anti‐AtpC (AS05 071), and anti‐RbcL (AS03 037).

### 4.14 Antibody Synthesis and Purification

The peptides (RLTS(p)VTLRPAASPSS‐Cys and RLTSVTLRPAASPSS‐Cys) for pSer8 were synthesized and conjugated to keyhole limpet hemocyanin carriers, and the polyclonal antisera were raised in rabbits (AtaGenix Laboratories Co., Ltd., Wuhan, PR China). pSer8‐specific antibodies were isolated by affinity chromatography using pSer8 peptides coupled to Cellufine Formyl, following Ishihama et al. (2014) [58]. The eluate was passed through an affinity column containing a nonphosphorylated peptide.

Anti‐OsAtpD1 antibody was produced using the full‐length protein synthesized by AtaGenix Laboratories Co., Ltd. (Wuhan, China).

### 4.15 PMF and ATP Synthase Activity Measurements

The PMF was measured using a Dual‐PAM‐100 (Walz, Effeltrich, Germany) equipped with a P515/535 emitter detector module [59]. ZH8015, *oscpk12*‐*cr*, *oscpk12*‐*cr*/OE‐*OsAtpD1^S8A^*, *oscpk12*‐*cr*/OE‐*OsAtpD1^S8D^*, and *RNAi*‐*OsAtpD1* were grown under natural conditions at 50 dps, which were dark‐adapted for more than 2 h, and the third upper leaves were illuminated for 10 min at light intensities of 843 *µ*mol photons m^−2^ s^−1^ red light. After illumination, the inverse electrochromic band shift kinetics were recorded for 1 min. The PMF values were calculated as previously described [59]. H^+^ conductivity through ATP synthase (g_H_^+^) was measured as described previously [20].

### 4.16 Chlorophyll Fluorescence Measurements

ZH8015, *oscpk12*‐*cr*, *oscpk12*‐*cr*/OE‐*OsAtpD1^S8A^*, *oscpk12*‐*cr*/OE‐*OsAtpD1^S8D^*, and *RNAi*‐*OsAtpD1* were grown under natural conditions. At 50 dps, the third upper leaves were used to measure chlorophyll fluorescence using an IMAGING‐PAM fluorometer (Walz, Effeltrich, Germany). The second leaves of ZH8015 and the *oscpk12*‐*cr* mutant were used to measure chlorophyll fluorescence after 5 days of HL and FL treatments. The NPQ was calculated as previously described [12]. Light intensity dependence of ETR(II), ETR(I), and NPQ was measured using an IMAGING‐PAM fluorometer with a series of light intensities (10, 36, 94, 214, 421, 611, 923, 1455, 2258, and 2805 *µ*mol photons m^−2^ s^−1^).

### 4.17 Statistical Analysis

All experimental data were analyzed and visualized using GraphPad Prism 9 (version 9.5.1). Data are presented as mean ± standard deviation (SD), with the sample size (n) of biological replicates clearly labeled within each figure legend for every independent comparative analysis. To evaluate the statistical significance between the two groups across multiple treatment conditions, a two‐way ANOVA followed by Fisher's least significant difference (LSD) post hoc test was used. Statistical significance was determined using a threshold of *p* < 0.05, with asterisks indicating varying significance levels: * (*p* < 0.05), ** (*p* < 0.01), *** (*p* < 0.001), and **** (*p* < 0.0001). For comparisons involving three or more groups, one‐way analysis of variance (ANOVA) was conducted, and Tukey's honest significant difference (HSD) post‐hoc test was applied for pairwise comparisons between two groups. A *p*‐value < 0.05 was considered statistically significant.

### 4.18 Accession Numbers

*OsCPK12* (LOC_Os04g47300), *OsAtpD1* (LOC_Os02g51470), and *UBQ10* (LOC_Os02g06640).

## Author Contributions

B.W., S.C., Q.L., and L.C. designed the experiments; B.W., A.C., Y.F., Y.W., P.X., Y.Z., X.Z., W.W., P.Y., D.C., J.F., Y.H., X.S., L.S., and A.Z. performed the experiments; B.W., A.C., and Y.F. analyzed the data; and B.W. and Q.L. wrote the manuscript. All authors read, revised, and approved the final manuscript.

## Funding

This project was supported by the National Natural Science Foundation of China (#32401794) and Basic Public Welfare Research Program of Zhejiang Province (Grant No. LY23C130003), the Talents Project of Zhejiang Province (2022R51009), the STI 2030‐Major Projects (2023ZD04066), the Agricultural Science and Technology Innovation Program of the Chinese Academy of Agricultural Sciences (CAAS‐ASTIP‐2013‐CNRRI), the Earmarked Fund for China Agriculture Research System (CARS‐01), the fundamental Research Funds and Central Public Welfare Research Institutions (CPSIBRF‐CNRRI‐202102), and High‐quality and resistant hybrid rice germplasm creation and new varieties development with international competitiveness (2022KJCX45).

## Conflicts of Interest

The authors declare no conflicts of interest.

## Supporting information




**Supporting File 1**: advs77268‐sup‐0001‐SuppMat.docx.

**Supporting File 2**: advs77268‐sup‐0002‐DataS1.xlsx.

**Supporting File 2**: advs77268‐sup‐0003‐DataS2.xlsx.

## Data Availability

The data that support the findings of this study are available from the corresponding author upon reasonable request.
