# Supplementary material for: OsCPK12‐OsATPD1 Module Regulates Photosynthetic Acclimation to Light Stress in Rice
Source: Adv Sci (Weinh). 2026 Sep 10:e77268. Online ahead of print. doi: 10.1002/advs.77268 (PMC13559773; doi:10.1002/advs.77268)
Supplement: Supplementary file 1 [file ADVS-9999-e77268-s003.docx]

**Supplemental** **Figure**

**Supplemental Figure S1.** The illuminance levels recorded under natural lighting (Control) and shading (Low light) conditions at various time points.

Data represent mean ± SD (n = 3 biological replicates). Experiments were repeated three times with similar results.

**Supplemental Figure S2.** Phenotypic characterization of *oscpk12*-*cr* plants under low light and five days after restoring normal light.

**Supplemental Figure S3.** The relative expression levels of OsCPK12 in ZH8015 leaves, measured at different time points under both control and low-light conditions.

Data represent mean ± SD (n = 3 biological replicates). The Ubiquitin gene was used as an internal control. Experiments were repeated three times with similar results.

 **Supplemental Figure S4.** Prediction results of the TargetP2.0 software and ChloroP1.1 Server for OsAtpD1.

(A) Prediction results from TargetP2.0 for OsAtpD1.

(B) Prediction results from ChloroP1.1 Server for OsAtpD1.

**Supplemental Figure S5.** Subcellular localization of OsAtpD1 in the protoplasts of ZH8015 and *oscpk12*-*cr*.

(A) Subcellular localization of OsAtpD1 in ZH8015 protoplasts.

(B) Subcellular localization of OsAtpD1 in *oscpk12*-*cr* protoplasts.

(C) Subcellular localization of OsAtpD1 by colocalization mCherry in rice protoplast of *oscpk12*-*cr*. Scale bars =20 *µ*m.

**Supplemental Figure S6.** Bimolecular fluorescence complementation (BiFC) assays demonstrated that the phosphorylation of OsAtpD1 at Ser8 did not affect the interaction between OsCPK12 and OsAtpD1.

OsCPK12-HYNE were transiently co-expressed with OsAtpD1^S8A^-HYCE or OsAtpD1^S8D^-HYCE in rice protoplasts, and after 24h transfected, fluorescence was observed using a laser scanning confocal microscope (ZEISS 700). OsCPK12-HYNE /GST-HYCE and HYNE/ OsAtpD1^S8A^-HYCE or OsAtpD1^S8D^-HYCE were used as negative control. Scale bars=2 *μ*m.

**Supplemental Figure S7.** Relative expression levels of *OsAtpD1* in leaves of ZH8015, *oscpk12*-*cr*/OE-*OsAtpD1^S8A^*, *oscpk12*-*cr*/OE-*OsAtpD1^S8D^*, *RNAi*-*OsAtpD1* and *oscpk12*-*cr* lines (n = 3 biological replicates for each genotype).

The values represent the mean ± SD. The Ubiquitin gene was used as an internal control. Letters above the bars indicate significant differences (*P* < 0.05). *P*-values were determined by one-way analysis of variance (ANOVA), followed by Tukey's multiple comparison test.

**Supplemental Figure S8.** Characterization of *osatpd1*-*cr* in the ZH8015 background.

(A) Phenotypes and genotypes of *osatpd1*-*cr* knockout lines. Bar=2 cm.

(B) The relative expression level of *OsAtpD1* in the leaf of ZH8015 and *osatpd1*-*cr* lines (n = 3 biological replicates). The Ubiquitin gene was used as an internal control.

(C) Quantification of thylakoid proteins in total proteins from ZH8015 and *osatpd1*-*cr* at the seedling stage*.*

(D) The activity of F1F0-ATP synthase in the leaves of ZH8015 and *osatpd1*-*cr* (n = 3 biological replicates).

(E) The content of chla and chlb in the leaves of ZH8015 and *osatpd1*-*cr* (n = 3 biological replicates).

Data in B and D-E represent mean ± SD. Letters above the bars indicate significant differences (*P* < 0.05). *P*-values were determined by one-way analysis of variance (ANOVA), followed by Tukey's multiple comparison test.

**Supplemental Figure S9.** The electric field component proton chemical gradient (ΔΨ) across the thylakoid membrane in the leaves of in leaves of ZH8015, *OsCPK12*-*OE*, *oscpk12*-*cr*/OE-*OsAtpD1^S8A^*, *oscpk12*-*cr*/OE-*OsAtpD1^S8D^*, *RNAi*-*OsAtpD1* and *oscpk12*-*cr* lines.

These were determined by the slowly relaxing phase of the ECS signal after illuminating the leaves with 843 *µ*mol photons m^-2^ s^-1^ red light for 10 min (n = 3 biological replicates for each genotype).The values represent the mean ± SD. Letters above the bars indicate significant differences (*P* < 0.05). *P*-values were determined by one-way analysis of variance (ANOVA), followed by Tukey's multiple comparison test.

**Supplemental Figure S10.** The relative expression levels of *OsAtpD1* in ZH8015 leaves at 50 days post-sowing (dps) were measured at different time points under both control and Low-light conditions. Data represent mean ± SD (n = 3 biological replicates). The *Ubiquitin* gene was used as an internal control. Experiments were repeated three times with similar results.

**Supplemental Figure S11.** Protein abundance of OsAtpD1 in total and chloroplast proteins extracted from the leaves of *oscpk12*-*cr*/OE-*OsAtpD1^S8A^* plants at different time points during the day. The values represent relative levels of the target protein normalized to the corresponding Actin or RbcL band. Experiments were repeated twice times with similar results.

**Supplemental Figure S12.** Relative amounts of ATP and NADPH amount in chloroplasts from leaves of ZH8015, *OsCPK12*-OE, *oscpk12*-*cr*/OE-*OsAtpD1^S8A^*, *oscpk12*-*cr*/OE-*OsAtpD1^S8D^*, *RNAi*-*OsAtpD1* and *oscpk12*-*cr* lines at 50 dps.

(A) Relative of ATP amount in chloroplasts from leaves of ZH8015, *OsCPK12*-OE, *oscpk12*-*cr*/OE-*OsAtpD1^S8A^*, *oscpk12*-*cr*/OE-*OsAtpD1^S8D^*, *RNAi*-*OsAtpD1* and *oscpk12*-*cr* lines at 50 dps (n = 3 biological replicates for each genotype).

(B) Relative of NADPH amount in chloroplasts from leaves of ZH8015, *OsCPK12*-OE, *oscpk12*-*cr*/OE-*OsAtpD1^S8A^*, *oscpk12*-*cr*/OE-*OsAtpD1^S8D^*, *RNAi*-*OsAtpD1* and *oscpk12*-*cr* lines at 50 dps (n = 3 biological replicates for each genotype).

All quantitative data were normalized to wild-type controls, with the average WT value defined as 1. Relative fold changes of genotypes were calculated from at least three independent biological replicates. Data are presented as mean ± SD. Letters above the bars indicate significant differences (*P* < 0.05). *P*-values were determined by one-way analysis of variance (ANOVA), followed by Tukey's multiple comparison test.

**Supplemental Figure S13.** Levels of thylakoid membrane proteins detected in the total proteins from leaves of ZH8015 and *oscpk12*-*cr* at 50 dps. The antibodies used for detection are indicated on the left.

**Supplementary Figure S14.** *OsCPK12* positively regulates rice yield-related traits.

(A) Seed setting rate of ZH8015, *OsCPK12*-*OE*, and *oscpk12*-*cr* mutant plants.

(B) Grain yield per plant of ZH8015, *OsCPK12*-*OE*, and *oscpk12*-*cr* mutant plants.

(C) 1000-grain weight of ZH8015, *OsCPK12*-*OE*, and *oscpk12*-*cr* mutant plants.

(D) Number of effective panicles per plant of ZH8015, *OsCPK12*-*OE*, and *oscpk12*-*cr* mutant plants.

Data are presented as means ± SD (n ≥ 10 plants per genotype). Letters above the bars indicate significant differences (*P* < 0.05). *P*-values were determined by one-way analysis of variance (ANOVA), followed by Tukey's multiple comparison test.
